# Revisiting applications of itaconic acid-based polymers obtained by (poly)condensation chemistry

**DOI:** 10.1039/d5gc06888f

**Published:** 2026-02-27

**Authors:** Nicola Bragato, Lazaros Papadopoulos, Andrea Pasquale, Sacha Pérocheau Arnaud, Minna Hakkarainen, Alessandro Pellis, Tobias Robert

**Affiliations:** a Fraunhofer Institute for Wood Research – Wilhelm-Klauditz-Institut WKI Riedenkamp 3 38108 Braunschweig Germany tobias.robert@wki.fraunhofer.de; b Department of Fibre and Polymer Technology, KTH Royal Institute of Technology Stockholm SE-100 44 Sweden; c Department of Chemistry and Industrial Chemistry, University of Genova via Dodecaneso 31 16146 Genova (GE) Italy; d Institute of Chemistry and Chemical Technology, Faculty of Natural Sciences and Technology, Riga Technical University P. Valdena str. 3 LV-1048 Riga Latvia

## Abstract

For more than a decade, itaconic acid (methylene succinic acid) has been one of the most promising bio-based platform chemicals. The reliable availability in the market, combined with a competitive price, makes the implementation of this molecule as a building block very interesting, also from a commercial point of view. In addition, itaconic acid being exclusively produced by biotechnological processes, is not a mere drop-in molecule. As it is not commercially available *via* classical chemical routes, it does not compete with the exactly same and, in most cases, cheaper petroleum-based building blocks, which is usually very challenging. But even more important, itaconic acid allows for novel chemistries and applications that cannot be achieved with the established petroleum-based building blocks. In this respect, itaconic acid has been used as an alternative monomer in radical polymerization. However, since itaconic acid is a dicarboxylic acid, it has also been explored in polycondensation reactions with a wide range of applications, spanning unsaturated polyester resins, coatings, medical applications and additive manufacturing. This work critically reviews the progress in itaconic acid-derived (poly)condensation polymers since 2016. In addition, the sustainability of this promising building block is discussed.

## Abstract 2

This review covers the recent progress in synthesis and appications of itaconic acid-derived monomers and polymers obtained by (poly)condensation chemistry.

## Graphical abstract

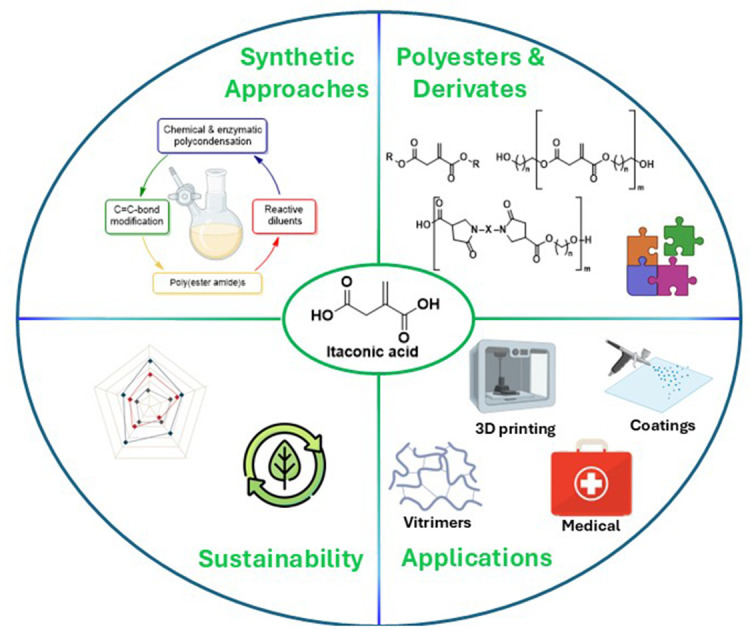

**Green foundation**

1. This review critically discusses recent advances of itaconic acid-based polymers prepared by polycondensation chemistry with examples in new fields of applications, such as additive manufacturing, covalent adaptable networks and biomedical applications. In addition, the sustainability of these materials is discussed by comparison of available LCA-data.

2. Itaconic acid is a very promising bio-based building block, as it can only be produced on a large scale by biotechnological processes. The use of itaconic acid for material applications shows how bio-based polymers can be a legitimate alternative to fossil-derived polymers with applications beyond the state-of-the-art.

3. First itaconic acid-based products are already commercially available. This review will show further potential of these polymers in a variety of applications and inspire future generations of green chemists to work on sustainable polymeric materials of this type.

## 1. Introduction

It is indisputable that polymers have become an indispensable part of our modern society with applications in almost every area of our daily life. However, it is also undeniable that the excessive utilization of polymeric materials has a huge negative impact on our environment, due to plastic waste and the excessive use of fossil building blocks for the preparation of the materials.^1^ One possibility to reduce the environmental impact of polymers is the replacement of monomers from petrochemical feedstock with those from renewable resources. In this respect, a range of bio-based building blocks have been in the focus of both academic and industrial research.^2–5^ In addition to the benefit of the bio-based origin and with that the potential of being more sustainable (not necessarily true in all cases), some of these building blocks are not commercially accessible from petrochemical feedstock These new monomers, which have not been previously exploited in polymeric materials, could enable novel polymeric architectures and subsequently new polymeric materials with unprecedented properties.

Itaconic acid (IA) or methylene succinic acid is a prominent example of this, as it was isolated as a by-product from the dry distillation of citric acid as early as 1836. However, this process is highly inefficient, and no other classical chemical syntheses have ever been commercially viable to produce IA on a large scale. On the other hand, biotechnological processes based on fungi, such as *Asperigillus terreus*, made itaconic acid commercially available in large quantities and at competitive prices.^6–8^ Today more than 100 000 t are being produced annually with a price of less than 2 € per kg.^9^ In terms of polymer applications, itaconic acid is a very versatile building block, due to its trifunctional structure and it has also been used in the ester form as a bio-based alternative to acrylates and methacrylates in radical (co-)polymerization reactions. In this respect, a lot of important research has been carried out, which was comprehensively reviewed very recently by Lienkamp and co-workers.^10^

Another important field of polymer application is polyesters and other condensation polymers derived from itaconic acid. The fact that itaconic acid exhibits two carboxylic acid groups, as well as an activated conjugated double bond, renders it a challenging, but also exceptional building block.^11^ It can be used to prepare unsaturated polyester resins (UPEs), similar to those derived from malic anhydride. However, the *exo*-position of the conjugated double bond makes it also reactive in UV-curing applications, similar to polyester (meth)acrylates that are usually utilized in such applications. Compared to saturated or “conventional” unsaturated polyesters, the synthesis of itaconic acid-based polyesters is more challenging due to several reasons related to the conjugated double bond present in the molecule.

**Scheme 1:**
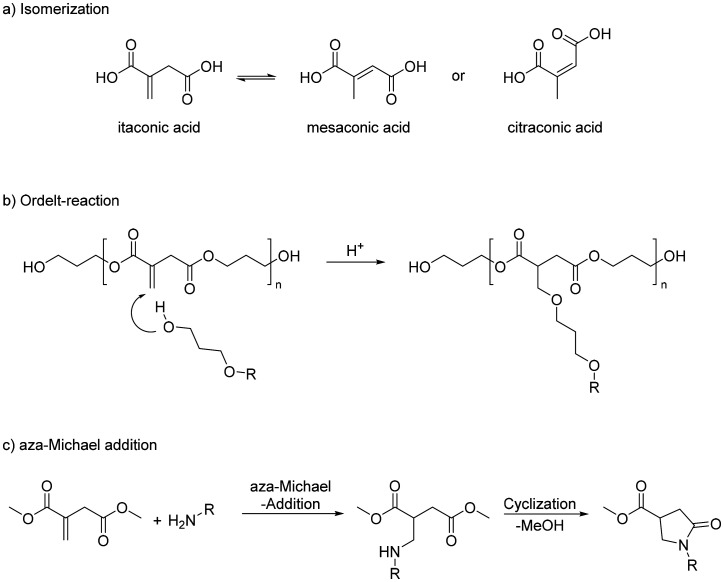
Undesired side-reaction during the (poly)esterification reaction of itaconic acid and itaconic esters.

**Scheme 2:**
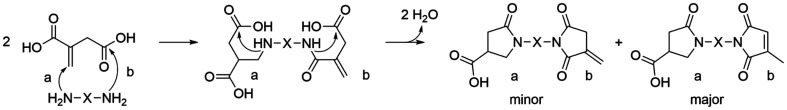
Undesired amidation reaction (b) combined with desired aza-Michael reaction (a). During the synthesis of bis-pyrrolidones, a nucleophilic attack by a second amine group on an itaconic acid carboxylic moiety can occur during the aza-Michael reaction (a) producing an asymmetric product.^32^ Adapted from ref. 32 with permission from Royal Society of Chemistry, copyright 2019.

1. Reactions at high temperatures (>150 °C) and long reaction times can lead to radically induced crosslinking of the itaconic acid moieties and crosslinking and gelation of the polyester. This can be suppressed by the addition of inhibitors, such as hydroquinone, methoxyphenol, phenothiazine, *etc*.

2. Due to the conjugated acid group, itaconic acid is less reactive compared to non-conjugated diacids, *e.g.* succinic acid or adipic acid, resulting in longer reaction time for the polycondensation reactions compared to that for other polyesters.

3. While the polycondensation reaction can be easily catalyzed by Lewis- and Brønsted-acids, the use of base-catalysts results in the isomerization of the double bond, forming significant amounts of mesaconic acid and smaller amounts of citraconic acid or their respective esters (Scheme 2a).

4. During the polymerization, the conjugated double bonds are susceptible to Ordelt-reactions (oxa-Michael addition) of diols. This leads eventually to the formation of undesired crosslinking and gelation of the polymer with pendant ether groups, typically of unsaturated polyester resins (Scheme 2b).^12^

5. The synthesis of linear unsaturated polyamides is not possible, as the amines undergo an aza-Michael addition at the conjugated double bond, resulting in the loss of the double bond. In the case of primary amines, the addition is followed by a ring-closing reaction to a 5-membered pyrrolidone ring. This reaction is known since the 1950s and was used to synthesize cyclic polyamides from itaconic acid (Scheme 1c).^13^

Despite these synthetic challenges, esters and polyesters from itaconic acid can be readily synthesized under appropriate experimental conditions. The synthesis can be conducted starting from itaconic acid or the corresponding esters. The latter are usually preferred, when higher molecular weights are targeted, which can be achieved by a standard two-step polycondensation reaction.^14^ Starting from itaconic acid directly on the other hand has several advantages. Firstly, the itaconic acid is significantly cheaper than the corresponding esters. Secondly, utilizing itaconic acid allows for the titration of the unreacted acid groups, which is a very convenient method to determine the conversion of the polycondensation reaction.^15^ Therefore, this kind of synthetic approach is preferred on an industrial scale and is usually used in resin applications, where the target molecular masses are below 10 000 g mol^−1^. Unsaturated polyesters from itaconic acid have high potential for use in a wide range of applications. The previous reviews on this class of polymers were compiled in 2016 and 2017 (Robert *et al.* and Kumar *et al.*).^16,17^ Since then, significant research efforts have been dedicated to this class of polymers and a lot of new research articles have been published. We are therefore convinced that a comprehensive review discussing research conducted in this field since 2016 is of high significance. Furthermore, this review will also give an overview of different monomers derived from itaconic acid, which were subsequently used as building blocks for polymers. First the synthetic approaches will be discussed, followed by the applications of these types of polyesters. In the end, a brief paragraph on the sustainability of these kinds of materials is presented followed by the conclusions.

## 2. Synthetic approaches

### 2.1 Chemical polycondensation of itaconic acid

Chemical polycondensations of itaconic acid-based resins have been conducted in the past similar to other types of polyester synthesis. Lewis-acid catalysts are usually being used for polycondensation reactions starting from itaconic acid esters, while polycondensation reactions from itaconic acid can be catalyzed by both Brønsted- and Lewis acids (Table 1). However, for a long time, no catalytic studies were reported in scientific literature. In 2017, Schoon *et al.* reported that the polycondensation of itaconic acid with 1,6-hexanediol did proceed smoothly with the Brønsted-acid catalyst methanesulfonic acid (MSA).^15^ However, they observed that a reaction of itaconic acid and 1,3-propanediol (1,3-PDO) under similar conditions led to incomplete conversions and eventually the gelation of the polyester resin. By conducting a small catalyst and substrate screening, they were able to conclude that reactions utilizing Lewis acids, such as Ti(OBu)_4_ and Zn(OAc)_2_, showed a higher substrate tolerance, while MSA led to competing ether formation, in the case of smaller diols, such as 1,3-PDO and 1,4-butanediol (1,4-BDO). Even more so, for the latter, the formation of THF occurs when Brønsted-acids are used, which is not observed with Lewis-based catalysts, especially with the water-tolerant Zn(OAc)_2_. In 2018, Brännström *et al.* also examined the influence of different catalysts on the co-polycondensation of dimethylitaconate with dimethylsuccinate and 1,4-butanediol.^18^ Again, the use of a Brønsted-catalyst – in this case H_2_SO_4_ or *para*-toluenesulfonic acid, respectively – led to THF-formation from 1,4-BDO. Base catalysts were also studied, which resulted in the isomerization of the conjugated double bond. The best results were obtained with either Ti(OBu)_4_ or an enzymatic catalyst. More details on the latter are discussed in section 2.2. Besides Ti- or Zn-based catalysts, the Sn-catalyst shows a very high reactivity. Here different comparably benign catalysts, such as stannous 2-ethylhexanoate (Sn(Oct)_2_) and commercial butylchlorotin dihydroxide, have been reported.^14,19^ However, Sn catalysts can be highly toxic, so it is important to avoid those containing tetrabutyl tin, which are particularly toxic.

**Table 1:** Overview of the catalysts used in polycondensation reactions with itaconic acid

Type of catalyst	Substrate scope	Temperature	Problems
No catalyst	No limitations	High	Long reaction times, low conversion
Brønsted acids	Not compatible with short chain diols	Medium (up to 130 °C)	Polyether/THF formation
Lewis acids	No limitations	High (>150 °C)	Toxic Sn-catalysts show highest activity
Enzymes	Not fully compatible with short chain diols	Low (<90 °C)	Limited substrate scope, low conversion

The group of Gadomska-Gajadhur recently reported a methodology to prepare itaconic acid-based polyester for tissue engineering applications. For this reason, they were interested in preparing the polyesters without the use of inhibitors. To investigate this reaction and optimize its conditions, they conducted a statistical approach applying the Box–Behnken plan using the reaction temperature, the amount of catalyst (Zn(OAc)_2_) and the reaction time.^20,21^ However, this method resulted in low conversion of the acid group and significant loss of double bond density due to undesired side reactions. While this might be tolerable for the desired application in tissue engineering, a high conversion of the acid group is usually desired in most applications in the coatings field. They subsequently used a similar approach synthesizing a copolyester with succinic anhydride as the second dicarboxylic building block. In this case the conversion of the carboxylic acid groups was slightly higher, but still not comparable to those in other synthetic methods usually applied.^22^

Kohsaka and coworkers developed a novel synthesis in which they used itaconic dichloride as a building block for polyesters.^23^ As standard chlorination methods utilizing thionyl or oxalyl chloride only led to poor yields and purities, due to the formation of the anhydride, they used a combination of dichloromethyl alkyl ether and a Lewis acid catalyst. The synthesis of the corresponding polyesters by melt condensation led to poor yields; however, interfacial polycondensations with bisphenol Z resulted in the successful synthesis of the semiaromatic polyester. While this method might not be generally applicable, especially on a larger scale, due to the use of chlorinated agents, it could be a valuable approach for very sensitive building blocks that do not tolerate high reaction temperatures and certain catalysts.

### 2.2 Enzymatic polycondensation of itaconic acid

Enzymatic syntheses of IA-based polymers have both advantages and drawbacks when compared to classic chemical syntheses, but they are a useful method that chemists can easily master and put to their use to process this thermally labile monomer. The catalyst of choice for the polycondensation of IA and its derivatives is the immobilized form of *Candida antarctica* lipase B (CaLB) better known as Novozym 435. This type of enzymatic catalyst combines the extraordinary thermal stability and broad substrate range of CaLB with the stability given by the solid support on which the enzyme is adsorbed, facilitating its separation from the reaction product and potentially its reuse in a new synthetic cycle.^24^ The enzymatic synthesis is usually carried out at temperatures between 50 °C and 100 °C in bulk systems in which the monomers (dimethyl itaconate and various aliphatic or cyclic diols) are heated to obtain a homogeneous melt that, after the addition of CaLB, is subjected to a 2-step process similar to the one reported for the metal- and acid-catalyzed polycondensation reactions.^18,25^ At these mild operational temperatures the authors of the various works did not observe any side reactions (see section 2.1 on chemical polycondensations), and the resulting polyesters presented a fully preserved vinyl group that is available for further post-polymerization functionalization.^25^ The only case where an undesired reaction occurred was when the enzymatic polycondensation of DMI and 1,8-diaminoctane was attempted. From the ^1^H-NMR spectra it was observed that the conjugated double bond and the amine functionalities reacted yielding a product with a pyrrolidone moiety integrated into its backbone, as described above.^26^ This ring closure reaction was exploited by Schulte *et al.* for the synthesis of bis-pyrrolidone structures that were then polymerized (using CaLB) with various aliphatic diols or used as additives for the plasticization of poly(lactic acid).^27^ Further work on these pyrrolidone-based polymers will be discussed in section 2.4. Despite the advantage of working both on IA esters and other bulkier IA derivatives, enzymatic catalysis has the drawback of yielding only short oligomeric chains having *M*_n_ < 5000 g mol^−1^. This is most likely because the conjugated acid group of IA is less electrophilic and in turn less reactive compared to non-conjugated carboxylic acid groups. In addition, the *exo*-position of the double bond brings some steric hindrance around the functionality, hindering the biocatalyst, which further reduces the reactivity of the conjugated acid group for enzyme-catalyzed esterification reactions.^25^

### 2.3 Unsaturated poly(ester amide)s from itaconic acid

As already described above, polycondensation reactions of IA with diamines to form polyamides with preservation of the α,β-unsaturated carbonyl group are not viable under standard condensation conditions and alternative routes must be envisaged. One possibility is the synthesis of poly(ester amide)s instead of polyamides by the reaction of preformed amide-containing building blocks that do not contain free amino groups. However, this makes the synthesis more complicated, and few examples of such polymeric structures have been reported to this day.

The first example of such IA-based poly(ester amide)s was reported by Ouhichi *et al.* by forming oligo amides from 1,6-hexane diamine and dimethyl adipate, together with ethylene glycol.^28^ These oligomers were in turn condensed with oligomers of IA and 1,6-hexanediol to form the desired poly(ester amide)s. An improved one-pot synthetic procedure was also described by the authors with the first step combining 1,6-hexane diamine with an excess of dimethyl adipate, before adding itaconic acid and 1,6-hexanediol, yielding resins with molecular weights up to 12 000 g mol^−1^. However, in both cases small amounts of free amines were still present in the final polycondensation step, which led in some cases to considerable base-catalyzed isomerization of the double bond. Nevertheless, these polymers could successfully be cured under UV-light despite viscosity and reactivity limitations compared to their polyester counterparts. To avoid the undesired isomerization reaction induced by the remaining free amines, Papadopoulos *et al.* used preformed amido diols that were obtained by the reaction of ε-caprolactone and 1,4-butanediamine or 1,6-hexanediamine.^29^ Reacting these amido diols with dimethyl itaconate, dimethyl sebacate and different diols, poly(ester amide)s could be synthesized without any detectable isomerization reaction of the conjugated double bond. The introduction of the amide group into these resins induced a thixotropic behavior, even at low amounts of amide groups in the resin. The materials were UV-cured by adding 5% of a photoinitiator resulting in rigid materials. A similar approach was later adopted by Vetri Buratti *et al.* where the same amido diols were utilized in combination with IA and vanillin.^30^ The obtained poly(ester amide)s displayed a low viscosity despite the presence of amides most probably due to the low *M*_w_ of 1600 g mol^−1^ resulting from the catalyst-free conditions.

### 2.4 Polycondensation polymers from itaconic acid under loss of the conjugated double bond

**Fig. 1:**
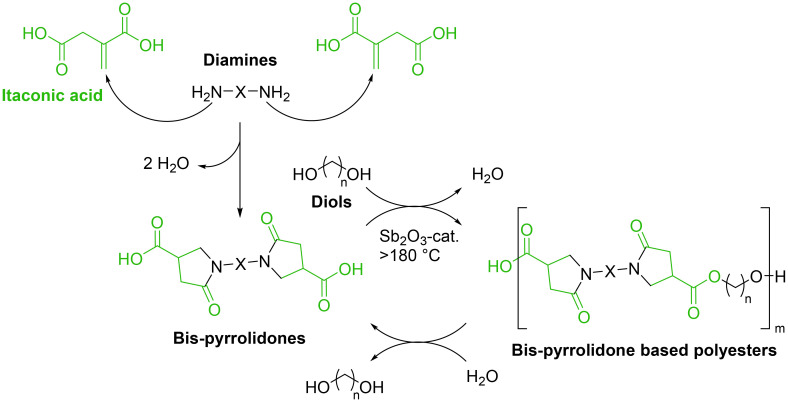
Synthesis of bis-pyrrolidones and their use as dicarboxylic acid building blocks for bio-based polyesters.^33^ Adapted from ref. 33 with permission from Royal Society of Chemistry, copyright 2016.

Considering the reactivity of conjugated double bonds, another possible IA derivatization approach is the reaction of this double bond with aliphatic, cyclic or aromatic diamines *via* an aza-Michael reaction followed by intramolecular cyclization (ring closing reaction) to yield bis-pyrrolidone dicarboxylic acid compounds (lactams).^31^ These interesting monomers were used as starting materials for the synthesis of different polyesters, semi-crystalline polymers and additives.^32^ Miller *et al.* synthesized a series of polylactam esters derived from IA, exhibiting higher glass transition temperatures and faster degradation rates compared to those of conventional bio-based polymers such as polylactic acid (PLA).^33^ IA was reacted with ethanolamine and ethylene diamine to produce the corresponding *N*-(2-hydroxyethyl)-2-pyrrolidone-4-carboxylic acid (HEPC), a racemic product, and bis(pyrrolidone carboxylic acid) (EBPC, Fig. 1). EBPC monomers were polymerized in a solvent-free system with several aliphatic diols, using antimony trioxide (Sb_2_O_3_, 2.0 mol%) as an optimized catalyst, resulting in a homologous series of polylactam esters (*M*_n_ = 19 500–24 900 g mol^−1^ by GPC). When performing homopolymerization, the best polylactam ester derived from HEPC was obtained using Sb_2_O_3_ as a catalyst, achieving a yield of 84%, a *M*_n_ of 20 800 g mol^−1^, a polydispersity of 2.1, and a glass transition temperature (*T*_g_) of 60 °C. Copolymerization of EBPC with different aliphatic diols exhibited the best result with 1,4-butanediol, using a diacid-to-diol molar ratio of 1.0 : 1.1. This resulted in a polymer with a *M*_n_ of 24 900 g mol^−1^, a polydispersity of 2.4, and a *T*_g_ lower than that of the homopolymer. Degradation studies conducted over one year revealed that exposure to air had negligible impact on *M*_n_. In contrast, exposure to water led to complete hydrolysis of the polymers into their monomeric components.

The synthesis of lactams *via* aza-Michael reaction can be carried out with high conversion under mild conditions using a small amount of solvent. Starting from dimethyl itaconate, the dimethyl ester derivative of IA, and combining it with aliphatic diamines of C4, C8, and C12 carbon chain lengths, it is possible to easily synthesize a series of bis-pyrrolidone-based structures (BPs). These compounds are used as monomers in combination with various diols in a polycondensation reaction catalyzed by CaLB, with the aim of synthesizing different polyesters and bis-pyrrolidone based additives. The best result in terms of oligoesters was an oligomer with an *M*_n_ of 3300 g mol^−1^ and a low dispersity value (*Đ* < 2). Considering the potential application of the bis-pyrrolidone-based additives, the shorter oligomers obtained from the polycondensation were used as plasticizers for PLA, improving the elongation at break up to 38%.^27^

Different combinations of bis-pyrrolidone monomers with 1,3-bis(4,5-dihydrooxazol-2-yl) benzene (IAox) and 2,5-bis(4,5-dihydrooxazol-2-yl) furan (FDCAox) were efficiently reacted by ring-opening polyaddition.^34^ The presence of dihydrooxazol-2-yl groups allows a 2-oxazoline ring-opening polyaddition between IAox and FDCAox and bis-pyrrolidone monomers producing 2-oxazoline resins, fully renewable and with tailored thermal properties.^35^ The undesired amidation reaction, which leads to the formation of an imide, could be the major byproduct in the bis-pyrrolidone synthesis with rigid cyclic diamines such as *trans*-1,4-cyclohexanediamine. During the aza-Michael addition, the second amino group can react with the carboxylic group at position four, followed by a ring-closing step that forms an imide, where isomerization of the double bond to an internal position has been observed (Scheme 2).^32^ Considering this specific problem, it was nevertheless possible to synthesize two different bis-pyrrolidones (BPDAs) with *trans*-1,4-cyclohexanediamine (*t*-CH) and *para*-phenylenediamine (*p*-Ph), and use them as starting materials for the polycondensation with various diol structures. In terms of polycondensation, the best results for poly([*p*-Ph-BPDA]-C10–12) and poly([*t*-CH-BPDA]-C6–12) were *M*_n_ = 40 400–46 300 g mol^−1^ and *M*_w_ = 87 700–110 600 g mol^−1^, respectively.

### 2.5 Modification of the conjugated double bond of itaconic acid

Regarding the modifications of IA-derived polymers, two main approaches can be distinguished: pre- and post-polymerization modifications of the monomer. In the pre-polymerization modifications, the double bond of IA is reacted to yield a more stable monomer for the subsequent polymerization process. For example, Giacobazzi *et al.* performed a K_2_CO_3_ catalyzed thia-Michael addition of 1-octanethiol on DMI (solvent: acetone, reaction time: 24 h) to yield 2-((octylthio)methyl)succinate.^36^ The derivatized IA, being a thermostable building block, was then polymerized with various aliphatic and cyclic diols leading to the production of polyesters having high molecular weights (up to 107 500 g mol^−1^ for BDO), homogeneous dispersities (proving their linear structure) and the absence of cross-linking. Similarly, another strategy reported the reduction of DMI to dimethyl 2-methylsuccinate and derivatization with isoprene to synthesize dimethyl 2-(4-methylcyclohex-3-ene)succinate (CS). These monomers were then used in a step-growth polymerization to synthesize polyesters with *M*_n_ values around 11 000 g mol^−1^ which were subsequently further derivatized to obtain both thermosetting and thermoplastic materials.^37^

**Scheme 3:**
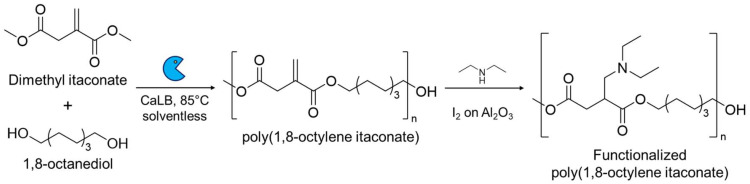
Enzymatic synthesis of poly(1,8-octylene itaconate) by using the lipase B from *Candida antarctica* (CaLB) as the immobilized biocatalyst in a solventless reaction system and the subsequent derivatization of the obtained functional polymer using diethyl amine with iodine immobilized on acidic alumina as the catalyst.

An alternative modification strategy is to polymerize IA (or its derivatives) exploiting the dicarboxylic functionalities using traditional catalysis (section 2.1) or biocatalysis (section 2.2) while preserving its vinyl group.^38^ After the functional polymer is synthesized several post-polymerization modifications can be carried out, for example, performing an aza-Michael reaction on an enzymatically synthesized poly(1,8-octylene itaconate) (POI) using diethyl amine and iodine on acidic alumina as the catalyst.^39^ This reaction yields an amine-pendant POI showcasing the efficiency of the catalyst in aza-Michael additions on bio-based itaconate polyesters, holding much promise in considerably reducing the prolonged reaction times typically used for these reactions (Scheme 3).

### 2.6 Monomers from itaconic acid

#### 2.6.1 Monoesters

**Scheme 4:**
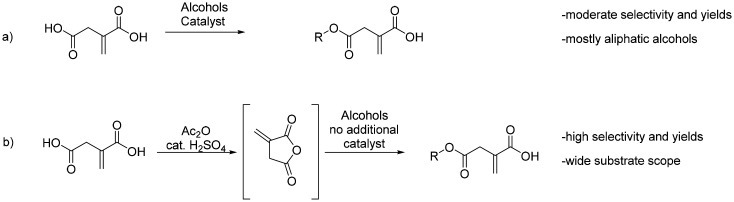
Overview of monoesters of itaconic acid reported; (a) obtained from itaconic acid and (b) from *in situ* prepared itaconic anhydride.^42^ Reproduced from ref. 42 with permission from American Chemical Society, copyright 2020.

Due to its three functional groups with one conjugated double bond and two carboxylic acid groups with different reactivities, IA is a valuable platform molecule to obtain various monomers. The overview of available monoesters from IA reported in the literature is displayed in Scheme 4. Traditional synthetic conditions to obtain such monoesters usually required the use of toxic catalysts or tedious purifications, while showing limitations regarding the alkyl group attached.^40,41^ Due to the conjugation of one of the carboxylic acid groups, IA exhibits a higher reactivity towards nucleophilic attack at the non-conjugated carboxylic acid. This advantage was used to develop a simple and robust one-pot synthetic pathway for itaconate monoesters by Pérocheau Arnaud *et al.*^42^ Itaconic anhydride was produced *in situ* and a large range of alcohols (Scheme 4) was added to obtain the β-monoesters in quantitative yields and with β/α selectivities up to 99/1, requiring only the evaporation of the produced acetic acid and excess alcohols as purification. Monomers of this type are good candidates for reactions with epoxides due to the conjugation of their remaining α-carboxylic acid. As such, they can be grafted on epoxidized vegetable oil like soybean oil, as described by Li *et al.*^43^ In this comparative study, renewable thermosets were produced from monomethylated itaconic acid to yield monomethyl itaconate, which was subsequently reacted with epoxidized soybean oil (ESO) *via* melt ring-opening esterification to yield monomethyl itaconated ESO (IESO). This bio-based resin was benchmarked against its acrylic analogue AESO. Isothermal thermogravimetric analysis revealed that monomethyl itaconate exhibited significantly lower volatility than acrylic acid, even at elevated temperatures (90 °C), supporting its role as a safer, greener alternative. Upon UV-curing with common reactive monomers, IESO-based systems demonstrated enhanced thermal and mechanical properties, including higher glass transition temperatures and E-modulus, as well as comparable or superior tensile strength and coating performance relative to the acrylic analogue AESO.^43^ Though they exhibit a lower reactivity towards UV-curing compared to their acrylate counterparts, the lower viscosity, higher bio-based content and higher carbon–carbon double bond conversion in the best cases proved that they could be promising alternatives.^42^

#### 2.6.2 Diesters

**Scheme 5:**
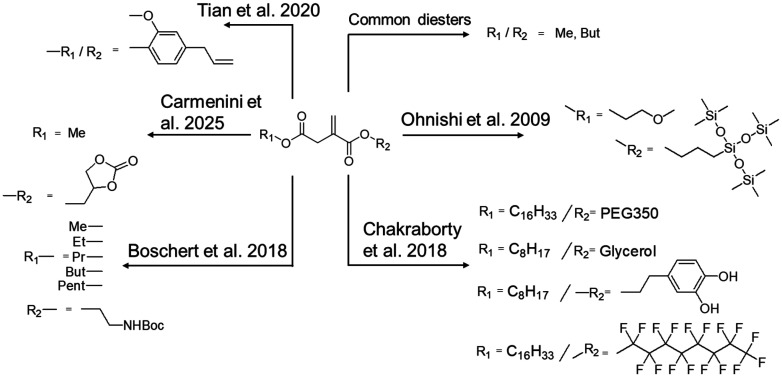
Overview of diesters of itaconic acid reported.

Diesters from IA can be used as (co)-monomers for radical polymerization reactions.^10,44–47^ Even beyond this application, these diesters are valuable monomers for transesterification reactions,^18^ where they can be used as monomers for polycondensation reactions instead of IA, either for reactions with less reactive alcohols or when milder conditions are required. They are also a greener alternative to acid halides. As such, diesters from IA represent valuable monomers for polymer chemistry. Diesters from IA for transesterification polymerizations are usually composed of methyl or butyl groups, like dimethyl itaconate or dibutyl itaconate, respectively.^48^ They are obtained by simple standard esterification in the presence of zinc acetate, tin octanoate or dibutyltin oxide.^49^ Boschert *et al.* reported the synthesis of asymmetrical disubstituted itaconate esters by reacting itaconic anhydride with linear alcohols (C1–C5).^50^ A protected amino alcohol was reacted with the obtained monoesters to form the asymmetrical amphiphilic diesters, which were then subjected to radical polymerizations. The resulting polymers were then used for antimicrobial applications. Similarly, Chakraborty *et al.* presented the synthesis of octyl or cetyl β-monoesters further reacted with activated polyethylene glycol, glycerol, fluoro alcohol or dopamine.^51^ This panel of amphiphilic diesters was radically copolymerized under UV-radiation, together with styrene to form surface-functionalized latexes. An amphiphilic itaconate diester with methoxyethyl and 3-[tris(methylsiloxy)silyl] propyl groups was also synthesized and crosslinked with styrene to form highly transparent membranes with good oxygen permeability to replace acrylated materials for contact lenses.^52^ Recently, Wang *et al.* explored the potential of dimethyl itaconate as a promising greener alternative to vinylene carbonate thanks to lower production cost and a synthetic route free of chlorinated moieties. Dimethyl itaconate was found to enhance battery performance and cycling stability, enlarging the scope of applications for such esters of IA.^53^ Degradable thermosetting polymers have been developed through thiol–ene click chemistry using IA-derived monomers combined with eugenol and this represents a promising way for using environmentally friendly materials in applications such as biomedicine and flexible electronics.^54^ Finally, Carmenini *et al.* recently synthesized glycerol methyl itaconate to increase the bio-based content of UV-curable resins, while enhancing the rigidity of soft resins or improving rigid resins’ elongation properties.^55^ The available structures of monoesters and diesters reported in the literature are shown in Scheme 5.

#### 2.6.3 Reactive diluents

Reactive diluents are small molecules that play a crucial role in UV-curing formulations as they are mixed with reactive oligomers to reduce the viscosity while enhancing the reactivity and reinforcing the 3-dimensional crosslinked network. They can represent up to 60% of UV-curing formulations. These monomers are to date almost exclusively composed of (meth)acrylate or acrylamide compounds.^56^ However, these small volatile molecules are known to exhibit strong allergenic and irritation potential.^57^ On the other hand, IA derivatives exhibit a much lower reactivity towards (UV-induced) radical crosslinking reactions due to the lower intrinsic reactivity of IA derivatives compared to (meth)acrylates. Furthermore, due the smaller functional leverage available on such small structures, performant alternatives to acrylates are difficult to develop. It is therefore of interest to improve the reactivity of these key molecules through innovative synthetic strategies.

**Fig. 2:**
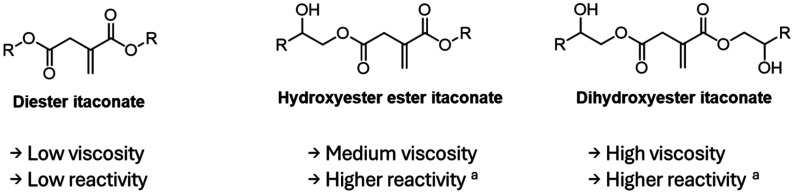
Three types of itaconic acid-based reactive diluents.^39^^*a*^Compared to other itaconate based reactive diluents. Figure created by the authors.

Using the different reactivities of its carboxylic acids, Pérocheau Arnaud *et al.* synthesized a set of itaconic acid-based reactive diluents with enhanced reactivities.^58^ Three types of monomers were synthesized, resulting in 13 new reactive diluents (Fig. 2). Diesters using linear and cyclic alcohols yielded monomers of low viscosity but relatively low reactivity towards UV-curing. Di-hydroxy esters displayed higher reactivities for itaconates (up to 4 fold compared to DBI's rate of polymerization) thanks to the reinitiation potential of the free hydroxy groups. However, such monomers were characterized by very high viscosities, limiting their use as reactive diluents in UV-curable formulations. Finally, the mixed ester and hydroxyester itaconates were proven most promising with a low viscosity and medium reactivity, especially when containing cyclohexyl groups. The best candidates were formulated with a bio-based crosslinkable resin between 10 and 50 wt% and allowed to lower the viscosity up to 20-fold. Despite significantly lower reactivity compared to those of acrylate reactive diluents, their introduction increased the final curing degree with final double bond conversion up to 11% higher, increasing the gel content from 72% up to 95%, and an improvement of the thermal properties. More recently, Maturi *et al.* formed difunctional reactive diluents by reacting dimethyl itaconate with 1,4-butanediol.^59^ These difunctional crosslinkers were mixed with poly(ester-thioether)s previously synthesized from IA and terpene-based diols. The introduction of such reactive diluents allowed the reduction of the inherent high viscosity of the resins while increasing the bio-based content up to 90 wt%. The addition of this reactive diluent together with a plasticizing agent allowed the reduction of the viscosities from 4.4–48.4 Pa s to 0.7–8.9 Pa s (at 25 °C) depending on the resin structure.

Dicks *et al.* synthesized sterically more demanding reactive diluents from castor oil and itaconic anhydride.^60^ These triglycerides were crosslinked with a standard unsaturated polyester synthesized from IA and 1,6-hexanediol (1,6-HDO), but the potentially high viscosity of the acid terminated reactive diluent required the addition of isobornyl methacrylate. The use of this reactive diluent with long aliphatic chains improved the miscibility of the formulation and the obtained printed materials showed a high level of details and good performances.

Finally, Niang *et al*. synthesized monomers from itaconic anhydride or IA and homocysteine thiolactone.^61^ The reaction of 1.1 equiv. of the protonated form resulted in a selective β-addition. The diamides could be obtained when 2.1 equiv. of the thiolactone were used. However, it should be noted that the pH had to be adjusted very carefully and in the case of the diamides the yields were below 20% even in the presence of coupling reagents. The monomers were then used as building blocks for both radical polymerizations as well as polycondensation coupled with ring-opening of the thio-ether.

#### 2.6.4 Functional monomers derived from the modification of the C=C double bond

**Scheme 6:**
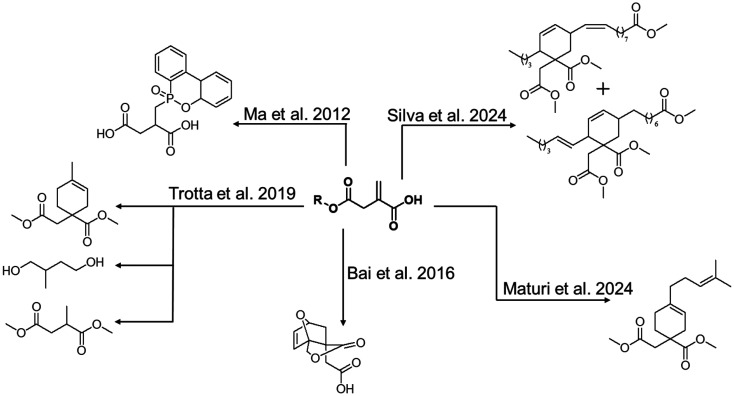
Overview of monomeric structures derived from itaconic acid after functionalization of the conjugated double bond.

Maturi *et al.* reported the formation of cycloadducts of dimethyl itaconate (DMI) to form myrcene-itaconate diester monomers to be copolymerized in standard unsaturated polyester resins (Scheme 6).^62^ Tailored reactive plasticizers and reactive diluents like 1,4-butanediyl bis-methyl itaconate were synthesized to tune the properties of final formulations, and materials ranging from very flexible to very rigid were obtained with a high bio-based content around 97%. With a similar approach, large monomers were synthesized *via* Diels–Alder reaction between DMI and methyl esters from tung oil by Silva *et al.*^63^ The resulting methyl esters were polymerized together with ethylene glycol or glycerol yielding novel bio-based polymers. Trotta *et al.* recently described the synthesis of saturated monomers from DMI, 2-methyl-1,4-butanediol and dimethyl(2-methyl succinate) by reduction using HSi(OEt)_3_ and hydrogen.^37^ These new monomers were polymerized together to form a thermoplastic polymer or combined with dimethyl 2-(4-methylcyclohex-3-ene)succinate, obtained from the Diels–Alder reaction of DMI and isoprene, yielding thermosetting materials. The obtained polymers with molar masses over 10 000 g mol^−1^ were also end-functionalized to allow further chain extension. Finally, an innovative monomer for ring opening metathesis polymerization was developed by Bai *et al.* from IA.^64^ This monomer was obtained through tandem Diels–Alder and lactonization between two bio-based building blocks: itaconic anhydride and furfuryl alcohol with a 100% atom efficiency, and can be combined with other ROMP polymers to tune the final properties.^54^ Ma *et al.* used itaconic acid to form green monomers with flame retardancy by combining it with 9,10-dihydro-9-oxa-10-phosphaphenanthrene-10-oxide.^65^ These monomers were successfully introduced in epoxy resins and showed to improve curing, thermal properties and improved flame retardancy. Zhao *et al.* also used these monomers in epoxy resins and further investigated their good flame retardant properties.^66^

## 3. Applications

### 3.1 Unsaturated polyester resins for thermosetting applications

Recent advancements in the development of unsaturated polyester resins (UPRs) based on IA reflect the growing interest in fully bio-based thermosetting polymers as sustainable alternatives to conventional petrochemical-derived materials. Since 2016, a number of studies have demonstrated the feasibility of synthesizing UPRs by replacing maleic anhydride with IA and using various bio-based reactive diluents (RDs), including dialkyl itaconates, guaiacol methacrylate, and 4-vinylguaiacol derivatives (Table 2).^67–72^ These developments address one of the main challenges in UPR formulation: identifying reactive diluents that combine low volatility, suitable viscosity, and high reactivity.

**Table 2:** Comparison of different UPE materials derived from itaconic acid

Name	Polyester composition			Cured materials				Ref.
	Diacid/ester (eq.)	Diols (eq.)	PE *M*_n_ (g mol^−1^)	Reactive diluents	*T*_g_ (°C)	*E* (MPa)	*σ* (MPa)	
UPR-DMI	IA (1 eq.)	1,2-PDO (1.05)	1324	DMI (40 wt%)	118	663	54	67
UPR-DEI				DEI (40 wt%)	104	496	48	
UPR-DiPI				DIPI (40 wt%)	96	390	33	
UPR-DBI				DBI (40 wt%)	65	268	21	
UPR:O-40	IA (0.5) + OA (0.5)	1,2-PDO (1.05)	1658–2015	DMI (40 wt%)	99	690	42	68
UPR:S-40	IA (0.5) + SA (0.5)				74	660	48	
UPR:A-40	IA (0.5) + AA (0.5)				73	700	52	
DMI90-DMI10	IA (0.5) + SA (0.5)	1,2-PDO (1.05)	1847	DMI(100–0%) + MMA(100–0%) (40 wt%)	60–79	490–860	—	69
PPI	IA (1)	1,3-PDO (1.2)	1100	GM (40 wt%)	142	2714	117	70
PPIF5-25	IA (0.75–0.95) + FDCA(0.05–0.25)		900–1100		119–128	2765–3521	115–123	
PPIF15S20-60	IA (0.25–0.65) + FDCA(0.15) + SA(0.2–0.6)		1100–1200		74–117	955–2460	42–13	
PISD30-100	IA(0.3–1.0) + SA(0.1–0.7)	1,3-PDO (1.2)	956–1430	AC4VG(40 wt%)	80–135	307–1022	27–48	73
S1-3	IA (0.25–0.75) + DDA(0.25–0.75)	1,3-PDO (1)	980–1600		0.5–19	20–196	5–31	74
UPE30-100	IA (0.1–1.0) + OA (0–0.9)	1,2-EG (1.04)	824–1448	DMI (40 wt%)	65–108	2062–3319	22–34	75
UPEF-I1-3	IA(0.25–0.35) + FDCA(0.15–0.25)	EG (0.15–0.25) + DEG(0.25–0.35)	3700–6200	DMI (40 wt%)	83–92	2300–3200	82–128	76
UPEF-SI1-3	IA(0.15–0.225) + FDCA(0.15–0.225) + SA(0.005–0.20)	EG (0.15–0.225) + DEG(0.275–0.35)	2000–4600		54–87	2100–3800	76–140	

**Fig. 3:**
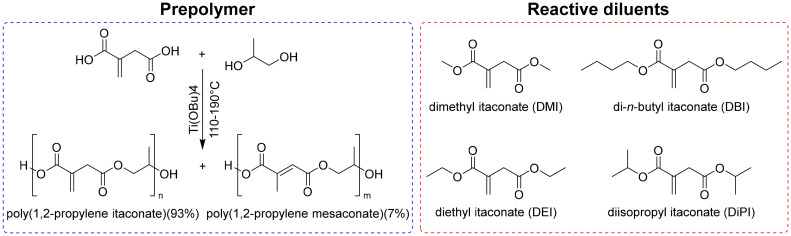
Titanium(iv) butoxide catalyzed polycondensation of itaconic acid and 1,2-propandiol with hydroquinone (as inhibitors [left] and as bio-derived reactive diluents [right]). Reproduced from ref. 67 with permission from American Chemical Society, copyright 2017.

Spasojevic and coworkers developed a one-pot synthesis method to produce 100% bio-based UPRs by melt polycondensation of IA with 1,2-propanediol (1,2-PDO), diluted with dialkyl itaconates such as dimethyl, diethyl, diisopropyl and dibutyl itaconates (Fig. 3).^67^ The resulting prepolymer exhibited an *M*_n_ of 1300 g mol^−1^ and an *M*_w_ of 3000 g mol^−1^. All IA-based reactive diluents exhibited significantly lower evaporation rates compared to styrene. In fact, after 15 hours at 30 °C, over 82% of the styrene evaporated, while the evaporation rate of DMI and diisopropyl itaconate (DiPI) was less than 10% in both cases, resulting in a significant reduction of VOC emissions. Differential scanning calorimetry showed that the glass transition temperatures (*T*_g_) of the cured resins ranged from 65 °C to 118 °C, depending on the reactive diluent used, with the styrene-containing system exhibiting the highest *T*_g_. The cured samples also showed moderate stiffness (270–660 MPa) and high break stress (21–54 MPa). The findings demonstrate that IA-based systems can match the thermal properties of traditional styrene-based UPRs, while significantly improving environmental and health aspects. This study shows the potential of fully itaconic acid-based UPR formulations. However, the lower reactivity compared to that of styrene-derived formulations is still a challenge, especially at short curing times usually applied in industrial manufacturing processes.

In a follow-up study, a series of fully bio-based UPR prepolymers were synthesized *via* melt polycondensation of IA with 1,2-PDO and various saturated dicarboxylic acids (oxalic, succinic, and adipic acids) and directly diluted with DMI (from 30 to 40 wt%).^68^ The resulting cured resins displayed tunable properties, with glass transition temperatures ranging from 60 to 97 °C and tensile strengths between 37 and 52 MPa. The elastic modulus, determined from tensile tests, ranged from 0.55 to 0.70 GPa, depending on the formulation and tensile strengths up to 52 MPa.

To overcome the lower reactivity, as well as higher viscosity of IA-based diluents, Spasojevic and coworkers examined the use of DMI in combination with methacrylic monomers such as methyl methacrylate (MMA) in UPR systems.^69^ The addition of MMA effectively leads to a significant reduction in viscosity, from 732 mPa s for the DMI-only formulation to 162 mPa s when pure MMA was used. However, this viscosity improvement came at the cost of thermal and mechanical performance since the glass transition temperature decreased from 79.3 °C to 60.4 °C, and mechanical and dynamic-mechanical properties dropped by 147% and 75%, respectively. However, the substitution of a small amount (10 wt%) of DMI with MMA caused a decrease in the resin viscosity of 30%, and a slight improvement of the mechanical and dynamic-mechanical properties. This balance suggests that partial substitution of bio-based diluents with small amounts of conventional monomers can enhance processability without severely compromising material integrity.

**Fig. 4:**
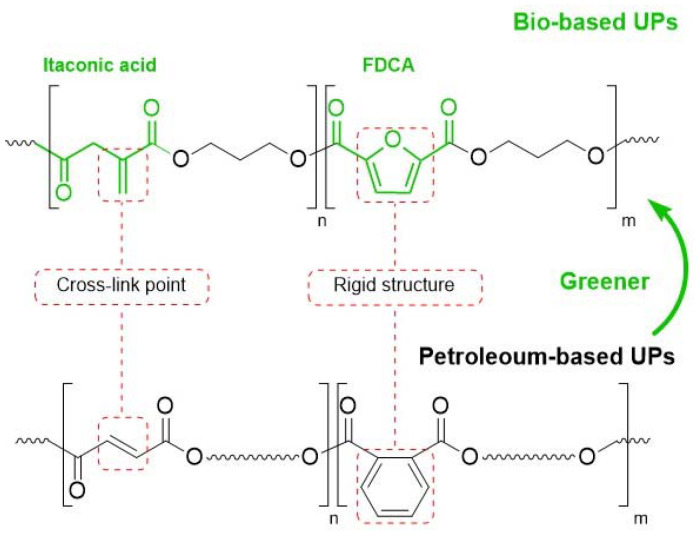
Fully bio-based UPs derived from 2,5-furandicarboxylic (FDCA), itaconic acid (IA), succinic acid (SA), and 1,3-propanediol (PDO). Reproduced from ref. 70 with permission from American Chemical Society, copyright 2017.

In another study, Dai *et al.* synthesized IA-based UPRs in which they incorporated 2,5-furandicarboxylic acid as a bio-based replacement for phthalic acid, which is often used in these systems to enhance the mechanical properties (Fig. 4). In addition, they also replaced styrene with methacrylic acid-based reactive diluents derived from guaiacol and ferulic acid, resulting in materials with high flexural strength, improved thermal stability up to 330 °C, and excellent glass transition temperatures above 130 °C.^70,73^

Furthermore, structural investigations of UPRs conducted by Molina *et al.* using techniques like ^1^H and ^13^C NMR and high-resolution mass spectrometry have revealed the presence of both linear and cyclic species in IA-based resins. These structural features, particularly those arising from the Ordelt reaction, can influence the curing behavior and final properties of the thermoset networks.^77^

Wong *et al.* developed a fully bio-based thermoset system based on poly(1,3-propanediol-*co*-1,12-dodecanedioate-*co*-itaconate) (PPDDI) as a sustainable alternative to traditional petroleum-derived thermosets.^74^ Synthesized *via* catalyst-free polyesterification using commercially available 1,3-PDO, 1,12-dodecanedioic acid (DDA), and IA, PPDDI oligomers exhibited uniform molecular weight distributions (*Đ* ≈ 2.0) with *M*_w_ values ranging from 2100 to 3400 g mol^−1^. The retention of unsaturated C=C bonds enabled free-radical cross-linking, and by varying the PD : DDA : IA molar ratios and dicumyl peroxide (DCP) content, a wide range of mechanical properties was achieved. The resulting materials ranged from flexible (UTS = 3.2 MPa, Young's modulus = 10.6 MPa, and elongation at break = 200%) to rigid (UTS = 31.0 MPa, Young's modulus = 196.4 MPa, and elongation at break = 64%). Increased IA content and longer curing times led to higher cross-link densities, reduced swelling, elevated *T*_g_, and suppressed crystallinity. Additionally, all formulations displayed hydrolytic degradability, tunable by IA content, supporting their promise as bio-based materials with adjustable end-of-life options.

Two recent studies illustrate complementary strategies for advancing fully bio-based composite materials with reduced environmental impact. Dai *et al.* synthesized a bio-derived UPR using IA, oxalic acid, and ethylene glycol. The polycondensation was conducted at 160 °C for 2 h under nitrogen flow, followed by 5 h at reduced pressure (0.08 MPa), using *p*-toluenesulfonic acid as a catalyst and hydroquinone as an inhibitor. The resulting low-molecular-weight terpolymer (*M*_w_ 820 to 1150 g mol^−1^) was formulated with dimethyl itaconate and *tert*-butylperoxybenzoate and used as matrix resin for a fully renewable composite with cotton fibers.^75^ The resulting composite material exhibited promising mechanical and thermal performance, with a tensile strength of ∼34 MPa, a *T*_g_ of ∼108 °C, and thermal decomposition temperatures ranging from 224 to 276 °C.

Savić *et al.* were able to modify the surface of cellulose nanofiber through a novel approach using DMI that acts simultaneously as an esterification agent and solvent.^78^ The resulting reaction mixture was used directly as a reactive diluent. The covalent incorporation of modified nanocellulose resulted in notable performance enhancements, including a 23% increase in tensile strength with 0.3 wt% of modified nanocellulose compared to that of the neat resin and elevated glass transition temperatures across all samples (*T*_g_ = 66–76 °C). Honzíček *et al.* investigated a novel chemical upcycling strategy for PEF.^76^ The polymer was depolymerized *via* solvolysis using diethylene glycol to produce hydroxy-terminated oligomers, which served as sustainable feedstocks for the synthesis of styrene-free UPR. These resins were prepared using IA as the source of unsaturation in the polyester backbone and DMI as a reactive diluent. The properties of the cured formulations were found to be strongly influenced by variations in the IA content within the polyester backbone, which governs the polymer network density.

In a more fundamental study Sbirrazzuoli and coworkers examined the curing mechanism of poly(hexylene itaconate) in the presence of a radical initiator.^79^ Both isothermal and non-isothermal conditions revealed that besides the radical crosslinking *via* the conjugated double bond, also other thermo-oxidative reactions take place during the curing process, leading to the formation of anhydrides and other recombination reactions and with that new crosslinks in the polymer network. These findings are of interest for a deep understanding of the radical curing of UPRs.

### 3.2 Coating applications

**Fig. 5:**
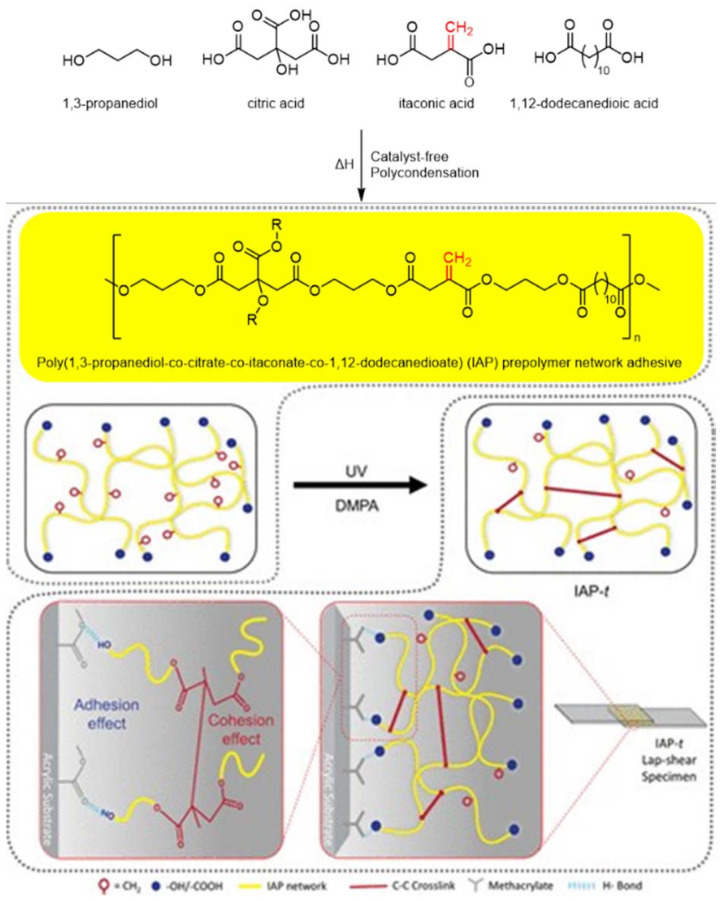
Schematic diagram of the synthesis of poly(1,3-propanediol-*co*-citrate-*co*-itaconate-*co*-dodecanedioate). Reproduced from ref. 82 with permission from American Chemical Society, copyright 2022.

Polyesters derived from IA have also been utilized in different applications in the coatings field, such as wood coatings, adhesives, printing inks, *etc*. The *exo*-position of the conjugated double bond also renders these bio-based UPRs suitable as binder resins for UV-curing coating applications, which is usually not possible with UPRs derived from maleic acid or maleic anhydride. Besides bio-based UPRs from IA, also polyester–urethane acrylate (PUA) systems incorporating IA have been successfully synthesized. For example, Patil *et al.* developed a UV-curable PUA resin using IA and 1,6-HDO to generate a polyol precursor *via* acid-catalyzed condensation.^80^ This bio-based resin was subsequently reacted with isophorone diisocyanate (IPDI) and 2-hydroxyethyl methacrylate (HEMA) to produce a UV-curable PUA system. When incorporated into conventional PUA formulations at varying concentrations, the IA-based PUA resin significantly influenced the rheological and thermal properties of the final coatings. Specifically, increasing the proportion of the bio-based component led to a reduction in viscosity, facilitating processing, while simultaneously enhancing rigidity. DSC and TGA analyses showed improved thermal stability and higher glass transition temperatures (*T*_g_ = 53–62 °C). Moreover, the IA-based systems demonstrated superior mechanical strength, chemical resistance, and solvent resistance compared to conventional PUA coatings. The coatings also exhibited favorable gel content and low water absorption, indicating good crosslinking efficiency and barrier properties. Furthermore, Feng *et al.* developed a solvent-free polycondensation of IA with glycerol and isosorbide yielding fully bio-based branched unsaturated polyester oligomers.^81^ The introduction of glycerol as a branching agent favored UV curing, resulting in coatings with higher gel content and crosslink density. These features translated into enhanced hardness, mechanical strength, solvent resistance, and excellent optical clarity, including high light transmission and low surface roughness, making the system particularly promising for transparent, durable bio-based coatings. The enhanced reactivity of IA-based UPRs allows for the development of (meth)acrylate-free binder resins for UV-curing applications. In this respect, Jamaludin *et al.* reported the synthesis of a fully bio-based branched itaconic acid-based polyester for UV-curing adhesive applications (Fig. 5).^82^ In this case, besides IA, and two bio-based diols, citric acid was used to introduce branching. The polycondensation reaction was conducted without the use of catalysts and with reaction times of less than 3 h. Neither the conversion of the polycondensation reaction nor the molecular weight of the polyester was characterized. However, the IR presented in the study shows the presence of both unreacted acid- and hydroxy groups, showing rather low conversions. Nonetheless, the polyester resin was formulated with a photoinitiator (2,2-dimethoxy-2-phenylacetophenone) and cured with UV-light. The resulting crosslinked polyester exhibited strong adhesion to a wide range of substrates. After a relatively long irradiation time of 30 minutes, the adhesive exhibited a shear strength of 1286 ± 19.2 kPa on acrylic substrates and showed similar high performance on stainless steel, wood, glass, and even low-surface-energy materials like PTFE. Notably, this IA-based adhesive also demonstrated robust underwater adhesion, enabled by a UV-triggered reduction in hydrophilicity that effectively repels interfacial water during curing. This capability was retained even under challenging conditions such as seawater, simulated body fluid, and silicone oil, highlighting the system's versatility for biomedical and marine applications.

IA-based polyesters have also been used as binder resins for UV-curing offset inks.^83^ Depending on the composition, polyesters with a bio-based content of up to 100% were synthesized. When co-formulated with acrylic acid-based binders, the curing reactivity was comparable to that of reference formulations. However, when the IA-based polyesters were used as the sole binder component, the reactivity was a little lower compared to that of the reference material. On the other hand, other properties important for UV-curing offset printing applications, such as misting and flow of the inks, were very similar to those of the reference ink showing the potential of this class of UV-curing materials derived from renewable resources also for printing ink applications.

As another example of completely acrylate-free UV-curing coatings derived from IA-based polyesters, Johansson and coworkers tested a series of polyesters derived from IA and 1,4-BDO, with different acid values (amount of unreacted acid groups), as well as a second series, where itaconic acid was partially replaced by SA.^84^ These resins were formulated with a photo initiator, applied on a glass substrate and cured by means of a UV-lamp. The properties of the resulting films were examined by DMA, pendulum hardness and micro indentation measurements. The resin consisting of only IA and 1,4-BDO and the lowest acid value (and in turn the highest molecular weight) showed the best coating performance. This can be explained by the higher density of the reactive double bonds, as well as higher initial molecular weight before radical crosslinking. The combination of both leads to a highly cross-linked network with good mechanical performance.

**Scheme 7:**
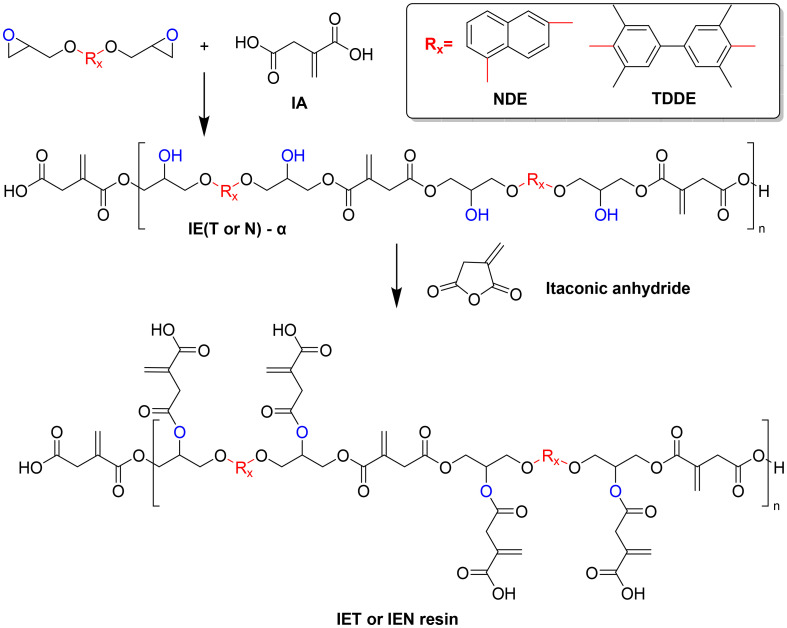
Synthesis of dual-phase systems by the reaction of epoxides with itaconic acid followed by a ring-opening of itaconic anhydride. Reproduced from ref. 85 with permission from Elsevier, copyright 2024.

Zhang and coworkers developed another acrylate-free coating system by first reacting itaconic acid with aromatic epoxides (1,6-naphthalene diglycidyl ether (NDE) or tetramethyl diphenol diglycidyl ether (TDDE)), followed by a reaction of the resulting OH-groups with itaconic anhydride (Scheme 7).^85^ These resins were then examined as dual-phase systems, where the coating film was first UV cured then followed by a thermal post-curing of the system. Both resins showed rather low double bond conversions after UV-curing (47 and 32%, respectively). The authors claim that the following thermal curing resulted in almost full conversion of the double bonds, which they proved using FT-IR spectra after thermal curing. Unfortunately, a direct comparison before and after the thermal curing is not shown to support that observation. The neat resins, as well as three different blends, were examined on their properties after curing, showing that some of the blends showed better mechanical performance compared to the neat resins.

IA-based polyester resins, such as pentaerythritol modified poly(butylene adipate-*co*-butylene itaconate)s have also been utilized in coatings for textile and biomedical uses.^86^ These copolyesters exhibited shear thinning behavior, excellent flexibility, low *T*_g_, and good adhesion to 3D mesh substrates, offering an alternative to traditional plaster coatings.

As another field of coating applications, IA-based unsaturated polyester resins derived from sebacic acid, glycerol and either 1,4-BDO or 1,6-HDO have been studied as wood coatings by Jagtap and coworkers, using partially bio-derived reactive diluents such as 2-hydroxyethyl methacrylate (HEMA), isobornyl methacrylate (IBOMA) and methyl methacrylate (MMA) as a replacement for styrene.^87^ These formulations showed thermal stability and performance comparable to commercial systems. Overall, itaconic acid proves to be a highly adaptable platform molecule for the development of bio-based coatings, adhesives, and surface treatments, offering sustainable alternatives to petrochemical systems without compromising material performance.

**Fig. 6:**
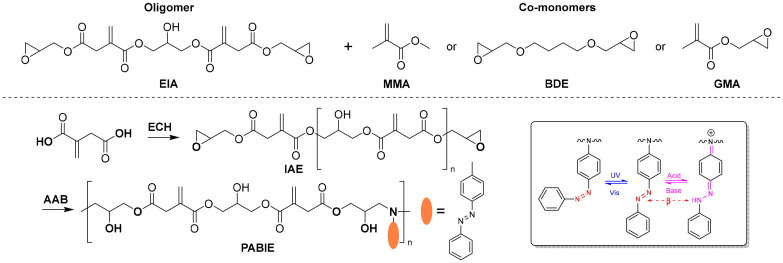
Synthesis of a trimer and an oligomer derived from the reaction of itaconic acid with epichlorohydrin and their use as dual-cure (top)^88^ or multi-responsive materials (bottom).^89^ Adapted from ref. 89 with permission from Springer Nature, copyright 2025.

Zhuo *et al*. recently reported a hybrid UV-curing coating derived from IA.^88^ On reacting IA with epichlorohydrin, a trimer (EIA) with both epoxy and conjugated double bonds was obtained, which can undergo both radical and cationic UV-induced crosslinking. Different formulations with methylmethacrylate (MMA), glycidyl methacrylate (GMA) and 1,4-BDO glycidyl ether (BDE) as reactive monomers (Fig. 6) were prepared and the properties of the resulting coatings after UV-curing were examined. Depending on the monomers used, the coating performance showed significant differences, with the formulation derived from glycidyl methacrylate outperforming the other materials. High conversions of the epoxy groups (98%) and conjugated double bonds (79%) were achieved and the resulting film exhibited a tensile strength of 26.5 MPa and a *T*_g_ of 55 °C. In addition, this film showed good gloss and lower shrinkage compared to the other films. Despite these good results, the synthesis of the oligomer relied on a 10-fold excess of the toxic and cancerogenic chemical epichlorohydrin. In this respect, an improved or at least a more efficient synthesis would be of high importance. A similar approach was used by Guo *et al.* to synthesize a precursor for a multi-responsive material.^89^ However, in this case an oligomer (IAE) derived from the reaction of IA with epichlorohydrin (ECH) was synthesized. By reacting this with an equimolar ratio of 4-aminoazobenzene (AAB), a linear epoxy resin (PABIE) with a *M*_w_ of 4000 g mol^−1^ is obtained. This oligomer is characterized by NMR, IR and GPC, which reveal the successful opening of the epoxide with the amine group (Fig. 6). However, also the double bond of IA is consumed, which can also lead to pyrrolidone formation as described in section 2.4. This is not at all discussed and leaves the actual structure of the polymer quite unclear. It is apparent in the NMR spectra and the IR spectra shown in the SI that the signal of the conjugated double bond does decrease significantly. Still, the material is indeed responsive to several triggers, such as UV-light, temperature and pH. While UV-light triggers the *cis*/*trans*-isomerization of the azobenzene moieties and with that the morphology, pH changes result in a significant color change of the polymer from yellow (basic) to purple (acidic), which opens a variety of applications of these materials, for example in the field of rewritable information storage.

A different approach employing epoxides in IA-based coatings was reported by Guigo and coworkers.^90^ They reacted IA with diglycidyl furfuryl amine (DGFA) to obtain epoxy resins that can be cured at ambient temperatures. The resulting coatings still exhibit the conjugated double bonds, which were exploited in a UV-induced thiol–ene reaction of fluorine-based thiols. The impact of this modification on the surface properties was investigated by contact angle measurements and XPS analysis, resulting in an increase of the contact angle from 56 to 99°.

### 3.3 Elastomers

**Scheme 8:**
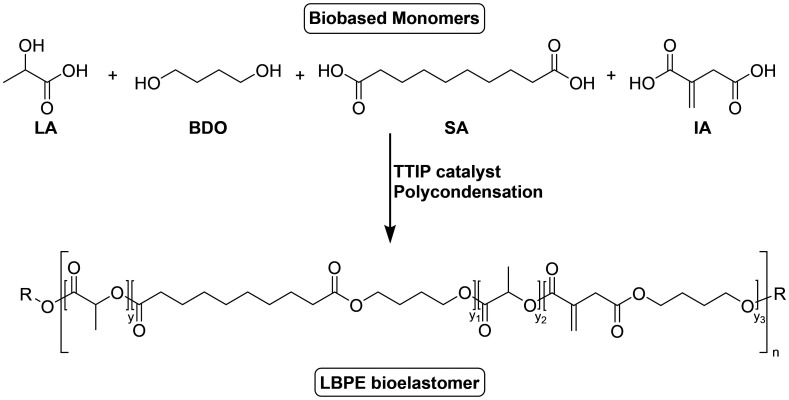
Synthesis of LA-containing itaconic acid-based polyester.^92^ Adapted from ref. 92 with permission from Springer Nature, copyright 2017.

Depending on the crosslink-density, polyesters from IA can also exhibit elastomeric properties. In recent years, several research activities have been reported towards IA-based elastomers not only due to their renewable origin, but also promising mechanical and thermal properties, which allow for the application in different fields, such as tire tread compounds, thermoplastic vulcanizates, flexible electronics, and high-performance rubbers. Most IA-based elastomers were synthesized by radical co-polymerization of diesters of IA with butadiene and other monomers, where an overview can be found in the recent work of Hao *et al.*^91^ However, also polyesters from IA have been utilized as elastomers. For example, Krishnan *et al.* developed a strategy to overcome the inherent brittleness of PLA-based elastomers while preserving the bio-content and the biocompatibility.^92^ For this they synthesized a polyester from IA, sebacic acid, 1,4-BDO and lactic acid (Scheme 8), which was blended with PLA. Dicumylperoxide was added as a radical initiator during the blending, resulting in a fully bio-based elastomer. The addition of the IA-based polyester led to a significant increase in impact strength (from 25 to 82 J m^−1^) and elongation at break (from 2.3 to 45%) when 20% of the IA-based polyester was used. SEM and DSC studies further confirmed this by revealing suppressed crystallinity. Importantly, the tunability of the mechanical response by varying the polyester content highlights the versatility of this approach, enabling a controlled transition from brittle to ductile materials.

Hu *et al.* developed a similar material by synthesizing a polyester from the same bio-based building blocks and blending it with PLA followed by a radical crosslinking induced by dicumyl peroxide.^93^ However, in this case, they studied the morphology and used the resulting thermoplastic vulcanizates in thermoplastic additive manufacturing. In addition, degradability and biocompatibility assessments underscored their potential for biomedical applications. Although modest reductions in performance were observed upon multiple reprocessing cycles, the materials retained acceptable mechanical integrity, highlighting their practical reusability.

Recently, Li and coworkers attempted to develop a bio-based elastomer through the synthesis of poly(1,5-pentylene itaconate-*co*-furanoate-*co*-ε-caprolactone) (PPeIFCL) copolyesters, designed to match or exceed the performance of petroleum-based rubbers and natural rubber.^94^ These aromatic–aliphatic polyesters, produced by transesterification and melt polycondensation of the bio-based monomers IA, FDCA, 1,5-pentanediol (1,5-PeDO), and PCL diol oligomers, followed by a crosslinking of the conjugated double bond of IA, show a very low crystallinity due to the “odd–even” effect of the monomers and in turn enhance the elasticity. The inherent 129.4° angle of the furan units, combined with asymmetric PCL segments and 1,5-PeDO-based chain irregularity, generates extensive chain entanglements, resulting in high tensile responsiveness. PPeIFCLs exhibit full amorphousness, high molecular weight (>28 700 g mol^−1^), excellent thermal stability (>327 °C), and progressive strengthening with increasing furan content, reaching 16.2 MPa tensile strength and 478% elongation. Mechanical characterization and low-field NMR confirm dense entanglement networks, while gas-barrier performance rivals that of commercial butyl rubber, showing the potential of IA-based elastomers.

### 3.4 Additive manufacturing

**Scheme 9:**
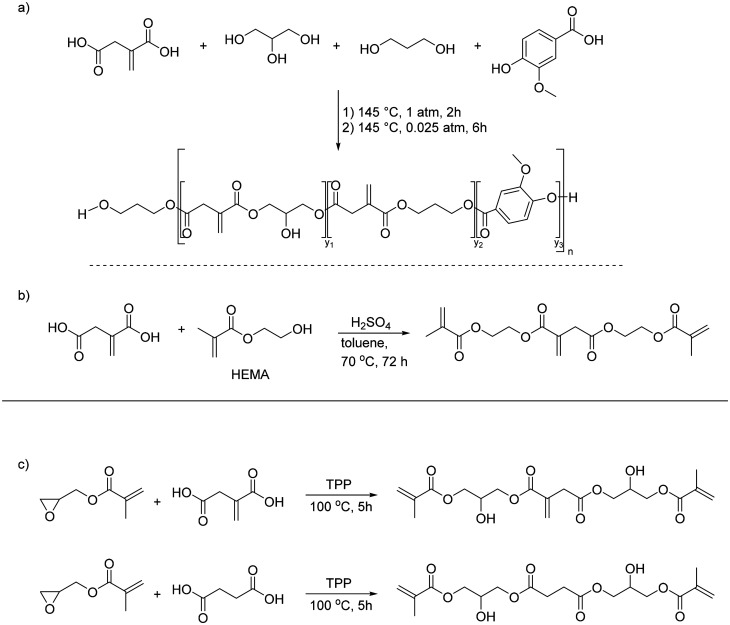
Synthesis of polymers and small UV-reactive molecules from itaconic acid. (a) Synthesis of a polyester;^95^ (b) reaction of itaconic acid with HEMA.^95^ Reproduced from ref. 95 with permission from Royal Society of Chemistry, copyright 2020. (c) Reaction of itaconic acid with glycidyl methacrylate.^96^ Adapted from ref. 96 with permission from American Chemical Society, copyright 2020.

As discussed in section 3.2, the *exo*-position of the conjugated double bond makes UPEs from itaconic acid more reactive compared to their counterparts derived from maleic acid. Therefore, they can be utilized as binder resins for UV-curing additive manufacturing materials. Franchini and coworkers reported the first example of this type of material in 2020.^95^ They synthesized a fully bio-based polyester resin from IA, glycerol, vanillic acid and 1,3-PDO (Scheme 9a). The polyester obtained was analyzed by GPC and showed low molecular weights, which can be expected due to the molar OH/COOH-ratio of 1.23. However, the authors report a very low polydispersity of 1.1, which is very unusual for polyester of this type, especially given the fact that they also use the trifunctional glycerol. Unfortunately, the GPC traces are not shown. In addition, the phenolic OH-group of the vanillic acid also suffers from low reactivity under standard condensation conditions, and the incorporation of this building block is most likely only due to the reaction of the acid group. The authors also synthesized small molecules by reacting IA and citric acid with two or three equivalents of HEMA, respectively (Scheme 9b).

**Fig. 7:**
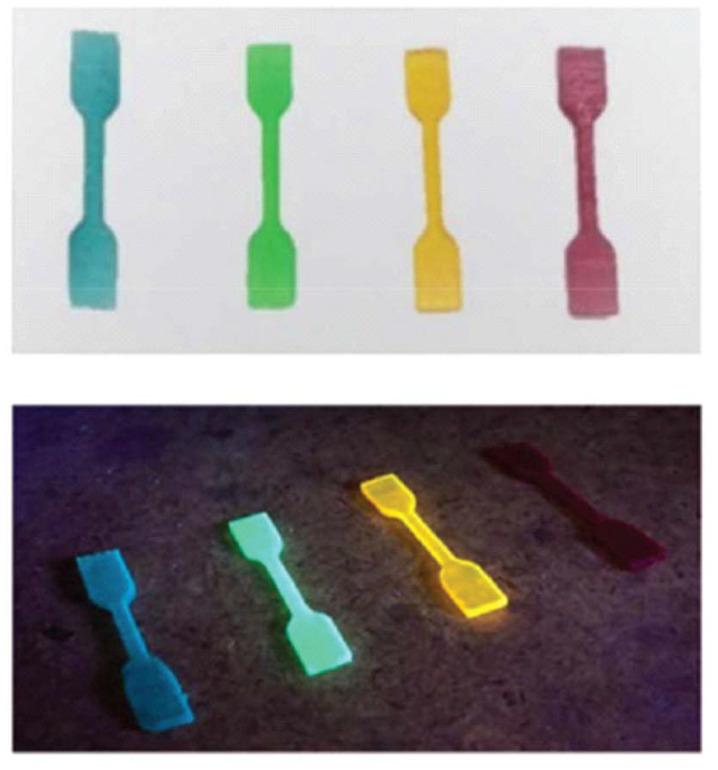
Bio-based phosphorescent parts prepared by additive manufacturing. Reproduced from ref. 95 with permission from Royal Society of Chemistry, copyright 2020.

By preparing formulations of these two sets of materials with photoinitiators and low amounts (0.01%) of dyes, phosphorescent parts in four different colors were manufactured on a DLP machine (Fig. 7).

A similar approach was used by Miao *et al.*^96^ However, they only utilized smaller molecules similar to those reported by Franchini and coworkers and not IA-based polyesters.^95^ In this case they reacted itaconic acid with glycidyl methacrylate to get trifunctional UV-curing building blocks (Scheme 9). In addition, they performed the same reaction with succinic acid, so they were able to study the influence of the conjugated double bond of the IA on the UV-curing crosslinking and the properties of the final materials. The parts printed from the IA-containing building blocks indeed showed better thermal and mechanical properties, showing the positive influence of the higher crosslink density due to the presence of IA. Based on the same monomer derived IA and HEMA, Ma *et al.* further explored formulations with other mono- and polyfunctional acrylates and analyzed their influence on the properties of the final cured parts.^97^

**Scheme 10:**
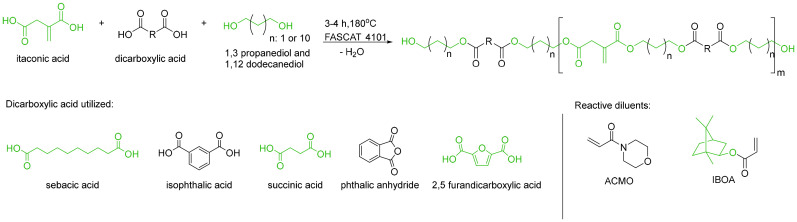
Polyesters from IA used as oligomers in UV-curing additive manufacturing. Reproduced from ref. 19 with permission from Elsevier, copyright 2023.

In 2023, Papadopoulos *et al.* examined the influence of different dicarboxylic acids on the properties of IA-based UV-curing additive manufacturing materials.^19^ For this, a set of different resins derived from IA, 1,3-PDO, 1,12-dodecanediol and a second dicarboxylic acid were synthesized and used as oligomers in UV-curing formulation with acryloyl morpholine (ACMO) and isobornylacrylate (IBOA, Scheme 10). As expected, the use of cyclic diacids resulted in higher E-modulus and *T*_g_ of the final cured part. Incorporation of the bio-based 2,5-furandicarboxylic acid led to materials with similar or even improved properties compared to the petrochemical phthalic anhydride or isophthalic acid. In consecutive studies, the authors studied the influence of the concentration and the composition of the reactive diluents on the UV-curing performance, as well as thermal and mechanical properties of the 3D-printed materials.^98,99^

A similar approach was undertaken by Jao *et al.*^100^ However, in this case they examined the influence of the diol structure of the IA-based polyester on the mechanical and thermal properties of the UV-cured AM-parts. Linear diols, such as ethylene glycol led to improved storage modulus and thermal stability, while the implementation of branched diols into the polyester resulted in increased ductility and impact resistance. As observed in the study of Papadopoulos *et al.*^19^ the incorporation of an aromatic dicarboxylic acid or in this case the ester (dimethyl terephthalate) had a significant influence on the rigidity and the tensile strength of the final materials. However, styrene was used as a reactive diluent in this case, which is rather unusual for UV-curing applications and makes a comparison with other materials based on acrylate or itaconate-derived diluents difficult.

Schlögl and coworkers compared four UV-curing resins with different bio-based contents in UV-curing additive manufacturing.^101^ They stepwise replaced petrochemical building blocks (cyclohexanedimethanol, 1,6-HDO, adipic acid, phthalic anhydride, and acrylic acid) with bio-based monomers (isosorbide, 1,3-PDO, sebacic acid, succinic acid, and IA). With this approach they were able to obtain a polyester resin with a high bio-based content of 95%. However, only one polyester was derived from IA, resulting in a resin with a very high viscosity (92 Pa·s compared to 1 Pa·s for the acrylic acid-based resin). This difference in viscosity makes the comparison with the acrylic acid-based resins of the study almost impossible, especially as no thermomechanical analysis was conducted on the IA-based material.

Another IA-based structure–property relationship study was reported by Chmely and coworkers.^102^ A polyester was synthesized from IA, succinic acid, 1,2-PDO, 1,4-BDO and 1,6-HDO with different ratios of the two bio-based acids. The resins were formulated with a difunctional acrylate (triethyleneglycol dimethacrylate) and a photoinitiator and subsequently cured in molds. In most cases, the E-modulus and *T*_g_ increased with higher itaconic acid content, due to higher crosslink-density without having a significant influence on the brittleness of the final materials. To examine the applicability of the formulations, the authors successfully tested the most promising material in UV-curing additive manufacturing, showing the potential of this type of bio-based material.

Dicks *et al.* used combinations of poly(hexamethylene itaconate), itaconated castor oil and isobornylmethacrylate (IBOMA) as UV-curing additive manufacturing formulations.^60^ The resulting cured parts exhibited good mechanical properties. In addition, the use of the itaconated castor oil facilitated the miscibility of the resins with IBOMA.

In addition to IA-based polyesters, other types of oligomers were also used in UV-curing additive manufacturing. Franchini and coworkers synthesized poly(ester amides) from IA, vanillic acid and preformed amido diols.^30^ They were utilized as oligomers in combination with small molecules tested in previous studies from the same group.^95^ The resulting UV-cured parts prepared by additive manufacturing were examined for their mechanical properties, which were similar to the results obtained from the polyester-based materials.

**Fig. 8:**
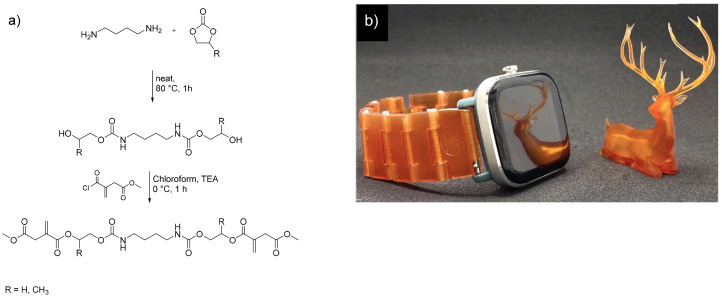
Synthesis from non-isocyanate urethanediol itaconates.^103^ Reproduced from the open-access journal *Progress in Additive Manufacturing*.

The same authors also reported the synthesis of UV-curing resins derived from non-isocyanate urethanediol itaconates.^103^ These resins are prepared by the reaction of preformed urethanediols with monomethyl itaconyl chloride (Fig. 8a). Eight formulations (bio-based contents from 30 to 90%) were prepared with different ratios of glycerol dimethacrylate, glycerol propoxylate triacrylate and a dimeric itaconate prepared from 1,4-BDO and DMI. All resins show a high E-modulus around 1 GPa with low elongations at break and detailed structures could be printed with the materials (Fig. 8b). However, long irradiation times up to 120 s were used.

In addition to the more established additive manufacturing procedures, such as SLA (stereolithography) or DLP (digital light processing), IA-based resins have also been used in a two-photon polymerization (2PP) process by Gontad *et al.*^104^ This allowed for the manufacturing of a small specimen with high lateral resolutions as low as 5 µm which cannot be obtained by standard UV-curing AM-processes.

**Scheme 11:**
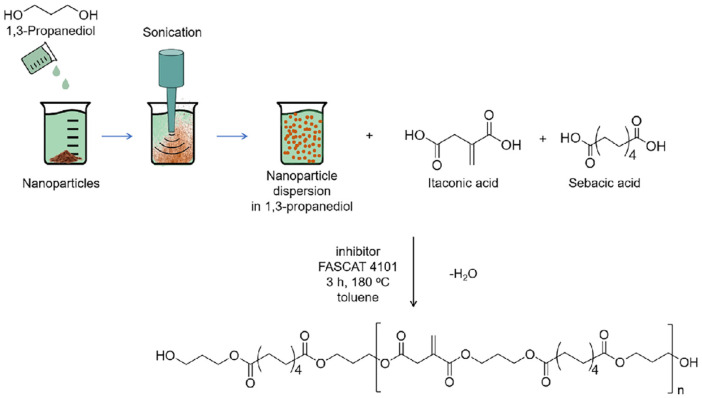
Synthesis of IA-based polyesters containing nanoparticles.^105^ Reproduced from the open-access journal *Giant*.

Another strategy to improve the mechanical and thermal properties of additive manufacturing materials is the incorporation of particles. However, in the case of UV-curable materials, this can be quite challenging, as the particles tend to agglomerate and settle over time, which could result in irregular distribution of the particles in the cured parts. To overcome this, Papadopoulos and coworkers used a strategy where a small amount of nanoparticles (0.2–1.0 wt%) was dispersed in 1,3-PDO, which was then directly used in the polycondensation reaction for the IA-based polyesters (Scheme 11).^105^ This indeed did prevent the agglomeration of the nanoparticles and the resins could be used to prepare different formulations with ACMO. Using this strategy, seven formulations were prepared with the following nanoparticles: crystalline nanocellulose, graphene, montmorillonite, and TiO_2_. All formulations were stable and reactive enough to be used in UV-curing additive manufacturing (5 s irradiation time per layer) and the resulting specimens were tested on their thermal and mechanical performance and compared to the virgin material without nanoparticles. Most of the nanoparticles had a detrimental effect on both curing reactivity and mechanical properties, due to lower light penetration, especially in the case of graphene and TIO_2_. However, the addition of nanocellulose had the opposite effect and resulted in higher UV-reactivity and improved mechanical properties. In addition, scanning electron microscopy coupled with energy dispersive spectroscopy (SEM-EDS) was performed and confirmed the even contribution of TIO_2_ particles in the printed samples.

**Scheme 12:**
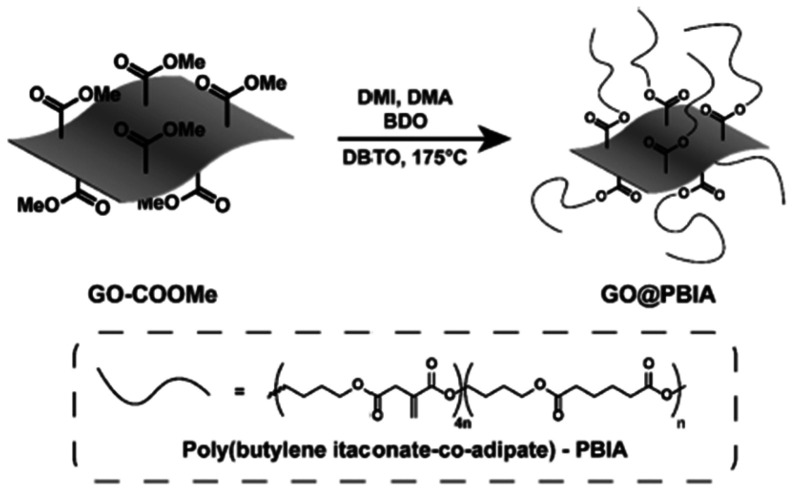
Schematic representation of the grafting of PBIA from the surface of carboxymethylated GO. Reproduced from ref. 106 with permission from American Chemical Society, copyright 2025.

In a very recent work of Maturi and coworkers, graphene oxide nanoparticles were modified and crafted with poly(butylene-itaconate-co-adipate) by the reaction of modified graphene oxide (GO) with DMI, dimethyladipate and 1,4-BDO (Scheme 12).^106^ This grafted graphene oxide was formulated with commercial UV-curing resins and test specimens were prepared by UV-curing additive manufacturing. The modification of the graphene oxide resulted in enhanced compatibility and dispersibility of the formulation and significantly improved mechanical properties even at very low loadings (0.05 wt%).

In a different approach, Dicks and coworkers also developed nano-reinforced AM-materials.^107^ However, they modified microcrystalline cellulose (MCC) with itaconic anhydride to obtain nanocrystals that could be covalently incorporated in the AM-material. The modified MCC was added to a 65/35 mix of itaconated castor oil (prepared in a similar fashion to the MCC) and either IBOA or IBOMA. The resulting materials showed improved mechanical properties when the modified MCCs were used. In addition, the materials exhibited shape memory behavior.

**Fig. 9:**
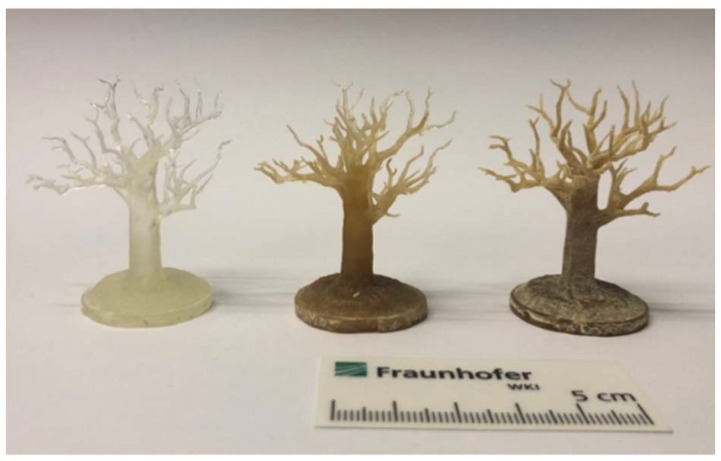
Printed parts from wood and olive kernel flour-based materials, neat material, materials with olive kernel flour and wood flour (left to right).^108^ Reproduced from the open-access journal *Reactive and Functional Polymers*.

Besides nanoparticles, larger particles can be used to improve the properties of UV-curing materials. In this respect, Robert and coworkers used wood and olive kernel flour in different amounts (0.5–15 wt%).^108^ The particles were mixed into the formulation of an IA-based polyester and ACMO as a reactive diluent and the stability of these formulations was monitored over time. Settling of the particles depended on the amount; however the formulations were stable enough for 2–3 h, so that test specimens could be prepared by means of additive manufacturing. The distribution of the particles in the cured parts was studied by light microscopy and was homogeneous even with 15 wt% of particles in the formulation. The addition of the particles resulted in an improvement of the tensile modulus and it was possible to prepare more complex structures from the formulations (Fig. 9).

**Fig. 10:**
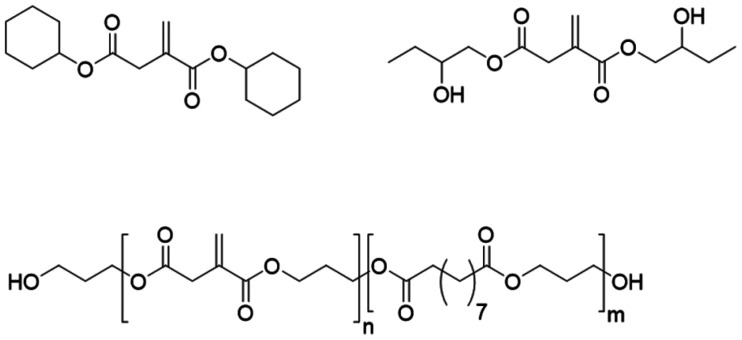
Itaconic acid-based monomers and PPIS used to prepare (meth)acrylate-free formulations.^58^ Figure created by the authors.

Even though all the examples described utilized IA-based small molecules or oligomers, in all formulations, (meth)acrylate-based materials were also part of the formulation, for example as reactive diluents. This is because IA exhibits a lower reactivity compared to its (meth)acrylic counterparts towards UV-induced radical polymerization. The combination with (meth)acrylates ensures that printing times can be in the same range as those of commercial materials in most cases (<10 s per layer). However, as methacrylic acid and acrylic acid are to date not available from renewable resources and possess in addition a much higher allergic potential compared to itaconates, it would be of great value to be able to design fully IA-based materials. The first example of (meth)acrylate-free radically curing additive manufacturing materials derived from IA was reported in 2021 by Robert and coworkers.^58^ As described in section 2.6.3 a set of different diesters were synthesized and tested for their UV-curing reactivity. The two most promising examples, dicyclohexyl itaconate and dihydroxybutyl itaconate, were then formulated together with poly(propylene itaconate-co-sebacate) (PPIS) and specimens were produced *via* additive manufacturing (Fig. 10). Due to the absence of (meth)acrylate-based reactive diluents, longer printing times (40 s instead of 3–10 s) per layer were needed. However, the parts exhibited a decent elastic modulus (up to 192 MPa at 25 °C), a high *T*_g_ (90 °C) and good thermal stability. These results were quite impressive, especially as the PPIS that was used as an oligomer in the formulation is a rather “soft” oligomer.

In 2023 Maturi *et al.* also reported UV-curing materials solely derived from IA-based materials.^59^ For this terpene-based diols were synthesized by thiol–ene click reaction of 2-mercaptoethanol with limonene, linalool, and geraniol. These diols were then incorporated into IA-based polyesters. Finally, these polyesters were formulated with a difunctional itaconic acid ester (1,4-butanediyl bis(methyl itaconate), BBMI) and a plasticizer. However, prolonged curing times per layer (2 min for layers of 100 µm) were needed for manufacturing the specimen.

**Scheme 13:**
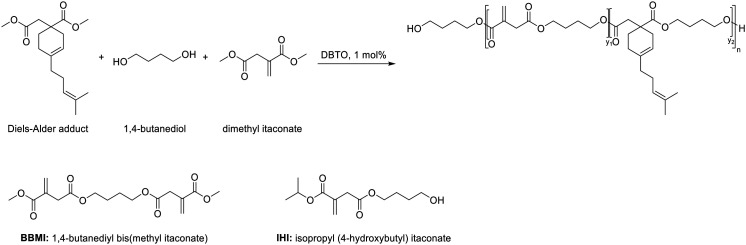
IA-based polyesters with dicarboxylic acids derived from the Diels–Alder cycloaddition product from itaconic acid and myrcene.^62^ Reproduced from the open-access journal *Additive Manufacturing*.

In a follow-up study, the same group prepared the Diels–Alder cycloaddition product of IA with myrcene, which was consecutively used as a dicarboxylic building block for polyesters (Scheme 13).^62^ From these esters, two different materials were formulated: a flexible one (with long chain methacrylates as diluents) and a tough one, which was formulated with BBMI and a monomeric itaconate (isopropyl (4-hydroxybutyl) itaconate, IHI) and a plasticizer. The curing times for these materials were again long (100 s per layer), but the authors were able to show that the composition of the formulation had a significant effect on the properties of the cured materials. In addition, the final parts had a very high bio-based content of up to 97%.

Recently, Franchini and coworkers synthesized a novel monomeric diester for additive manufacturing derived from IA by reacting monomethyl itaconate with oxalyl chloride, which is subsequently quenched with glycerol carbonate to obtain glycerol carbonate methylitaconate (GCI).^55^ They were able to show that they could use up to 70% of this monomer in combination with monomeric acrylates in UV-curing AM-formulations. Despite promising properties, the parts had to be cured with long irradiation times up to 120 s.

A slightly different approach for IA-based UV-curing additive manufacturing materials was undertaken by Heise and coworkers.^109^ They synthesized polyesters from 1,4-BDO and caprolactone by ring-opening polymerization followed by an end-capping with IA. The synthesis was conducted without solvents; however, it is not specified how the water which formed in the condensation step of the IA was removed, as solvent-free polycondensations are standard reactions and are conducted under reduced pressure. Subsequently this acid-terminated polyester was formulated with pentaerythritol tetrakis(3-mercaptopropionate), a thiol-based crosslinker, a photoinitiator and dioxane or γ-valerolactone as non-reactive solvent. This was necessary due to the difference in the solubility of the compounds. Even though they printed hydrogels, the materials did not allow for a solvent-free UV-curing AM, which is usually performed. They were able to show that they could not only use IA as a crosslinking moiety, but the free carboxylic acid groups of IA allowed for the preparation of pH-responsive materials. In addition, the end-of life options of the materials were also examined, showing that the polymers could be hydrolyzed with the option to reuse the bio-based monomers.

### 3.5 Covalent adaptable networks

IA and itaconic anhydride are interesting monomers for the preparation of covalent adaptable networks (CANs) that can bridge the properties of thermosets with the processability of thermoplastics. Most IA-based CANs reported so far are associate CANs (vitrimers) and based on transesterification exchanges. Epoxy-chemistry is by far the most utilized, but polyurethane, phosphate, boron-ester and disulfide-based CANs are also reported. In some cases, only self-healing properties are reported, while other studies also demonstrated mechanical recyclability and in fewer cases chemical recyclability. Many of the reported materials exhibit additional value-added properties such as flame retardance, UV-blocking or fluorescence properties. Often extra functionality is obtained by utilizing the double bond of itaconic acid for *e.g.* thia-Michael addition or phosphorus addition. The aimed applications vary from free-standing films to coatings, adhesives, foams, sensors, 3D-printing materials and fiber reinforced composites.

**Scheme 14:**
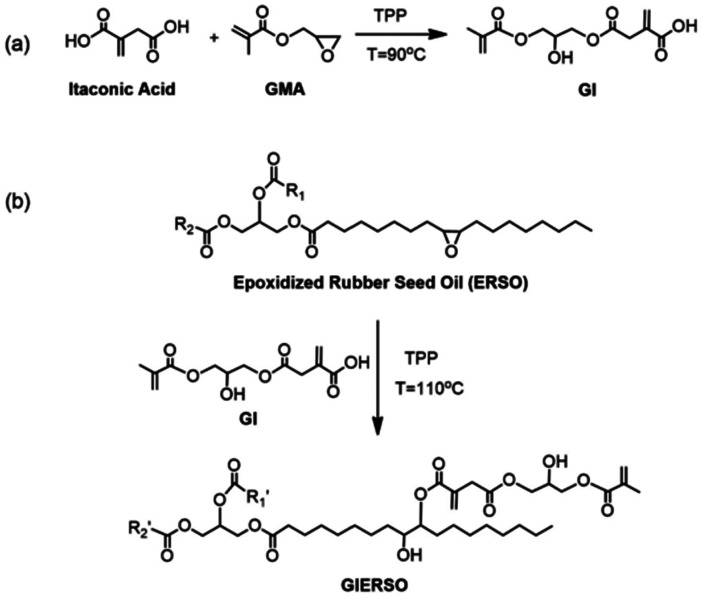
Synthesis of vegetable-oil based itaconate/methacrylate building blocks used in vitrimer applications. Reproduced from ref. 110 with permission from Elsevier, copyright 2021.

Majority of the studies on itaconic acid-based vitrimers selected transesterification as the dynamic chemistry. Epoxidized vegetable oils offer a substrate of high bio-based content, and the ring opening with itaconic acid further enhances the renewability of the materials. Materials based on rubber seed oil,^110^ linseed oil,^111^ castor oil,^112^ and soybean oil^113^ have been described (Scheme 14), targeting applications from coatings with UV-light blocking abilities to reactive extrusion to produce PBAT based foam. Cardanol has also been used to prepare vitrimers, acting as another bio-based substrate for recyclable materials.^114^

However, the usefulness of IA extends beyond the role of the renewable crosslinker. The presence of the pendant double bond enables the synthesis of modified curing agents that can provide additional functionality to the final material. The most prominent example is flame retardancy, which is achieved through the synthesis of DDP – a phosphorus-functionalized IA-derivative formed *via* the addition of DOPO (9,10-dihydro-9-oxa-10-phosphaphenanthrene-10-oxide) to the double bond of IA. This concept has been explored across different epoxy substrates,^115–117^ as well as in the preparation of fiber-reinforced composites.^118^ By adjusting the phosphorus content the resulting materials can display self-extinguishing properties and reduced smoke release while retaining their mechanical recyclability. Moreover, in the case of the composites, aminolysis has also been reported as a way of recovering carbon fibers from the cured network. These studies highlight the key role of IA in the development of vitrimers with both high renewable content and enhanced flame-retardant behavior – a combination of properties that addresses the dual demands of sustainability and material safety.

**Scheme 15:**
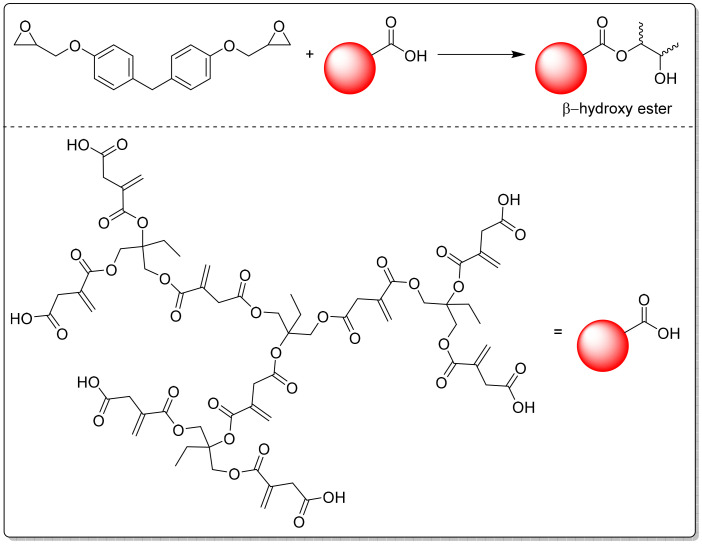
Branched polyester derived from glycerol and itaconic acid with a high amount of free carboxylic acid groups and its reaction with a diepoxide. Reproduced from ref. 120 with permission from Royal Society of Chemistry, copyright 2022.

The utilization of IA is not restricted to its role as a curing agent. In several studies, IA was integrated into the main polymer backbone. This can be achieved *via* the synthesis of branched polyesters that exhibit a high amount of free carboxylic acid groups along the chain, which usually stem from multifunctional monomers like glycerol,^119^ 1,1,1-tris(hydroxymethyl)propane,^120^ and similar polyols (Scheme 15). Another strategy that has been employed is the preparation of macromonomers from the opening of oxirane rings, which results in β-hydroxy groups which are highly active towards transesterification reactions.^121,122^

IA-based epoxy resin has also been used for the preparation of a phosphate based vitrimer, which was cured with phytic acid.^123^ In this system the excess of P–OH groups facilitated the exchange reaction and led to a recovery of 80% of the tensile properties after thermal reprocessing, and simultaneously it improved the flame retardancy properties. The same IA-based epoxy resin was employed in a follow-up study to produce carbon fiber composites, where it was combined with glycerol and maleic anhydride.^124^ The resulting thermoset composites could be reprocessed in a hot-press at 190 °C and they recovered close to 100% of the mechanical properties when an equimolar ratio of maleic anhydride and glycerol was employed. Furthermore, the polymer matrix could be degraded when immersed in basic media to recover the carbon fibers when the material reached its end of life. Finally, the C=C bond of IA-based monomers can also be utilized to prepare a macromonomer through radical polymerization, where the dynamic nature is derived from the alkyl groups of the monomers employed.^125^ In this study, the properties of the recycled materials could be adjusted based on the content of OH terminated monomers in the material.

As demonstrated by the studies cited herein, the presence of a catalyst is crucial for these dynamic networks which are based on transesterification reactions, as its absence can lead to significant deterioration in mechanical properties.^111^ Among the most widely used catalytic systems are Zn-based catalysts and triazabicyclodecene (TBD), each operating through distinct mechanisms. Zn-salts function as Lewis acids, coordinating with the ester carbonyl and thereby increasing its electrophilicity and facilitating nucleophilic attack by hydroxyl groups. TBD, on the other hand, acts either as a strong organic base, deprotonating alcohols, or as a nucleophilic catalyst capable of transiently interacting with the ester group.^126^ While TBD is generally considered the more catalytically active species, its thermal stability is limited. This is particularly important in vitrimeric systems, where prolonged exposure to elevated temperatures is often required to enable effective stress relaxation and reprocessability. Under such conditions, TBD may undergo partial degradation, potentially compromising the long-term performance of the material. In contrast, Zn-based catalysts offer greater thermal robustness, making them more suitable for applications involving sustained high-temperature processing. As a final comment, TBD has been found to act as a radical scavenger, which reduces the reactivity of systems where the formation of radicals is the main crosslinking mechanism.^127^ Zn-salts do not display similar behavior and therefore can be preferred for specific applications, such as UV curing or 3D printing.^128^

**Scheme 16:**
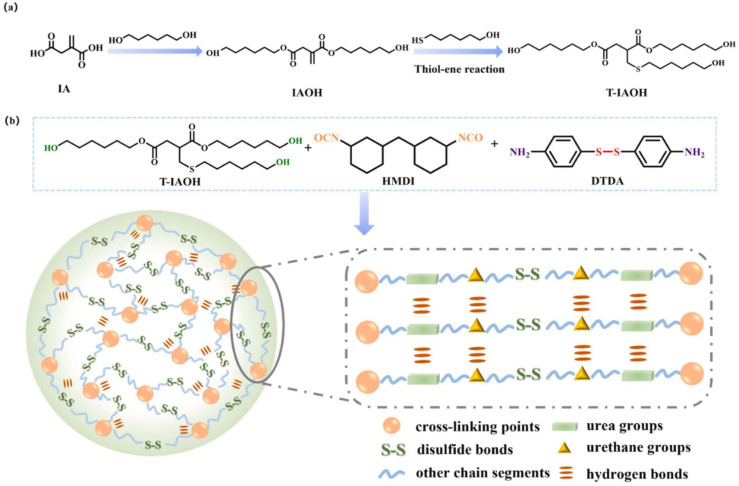
Synthesis of crosslinked dynamic covalent networks based on itaconic acid-based polyurethanes. Reproduced from ref. 130 with permission from Elsevier, copyright 2024.

Another important class of IA-based dynamic polymer networks leverages disulfide bonds to enable reprocessability and self-healing behavior. The disulfide linkage is well-studied and offers several advantages, including relatively fast exchange kinetics under mild thermal or chemical conditions and compatibility with a wide range of polymer matrices.^129^ A notable practical benefit is the availability of commercially accessible disulfide-containing monomers, which facilitates straightforward formulation and scalability (Scheme 16).^130,131^ This synthetic accessibility has also enabled the design of hybrid systems incorporating dual exchange mechanisms – most commonly disulfide metathesis in combination with transesterification – which have been shown to exhibit rapid and efficient stress relaxation.^132,133^ Interestingly, in recent studies, the direct vulcanization of IA has been demonstrated, to create disulfide linkages, and the subsequent crosslinking of epoxy monomers, yielding polymeric and composite materials with promising mechanical and reprocessing properties.^134,135^ These findings highlight the versatility of disulfide chemistry not only in standalone dynamic systems but also as a modular tool for tuning the vitrimeric behavior of hybrid systems.

Dioxaborolane metathesis has also been used in a couple of studies on IA based CANs. These systems exhibit fast relaxation and very high recovery of the mechanical properties after the reprocessing. Interestingly, both studies targeted adhesion applications of their materials, and the results on metal substrates were very promising.^136,137^ Moreover, the latter study is another example of a dual mechanism CAN, as boron esters were combined with traditional transesterification on carboxylate esters.

**Fig. 11:**
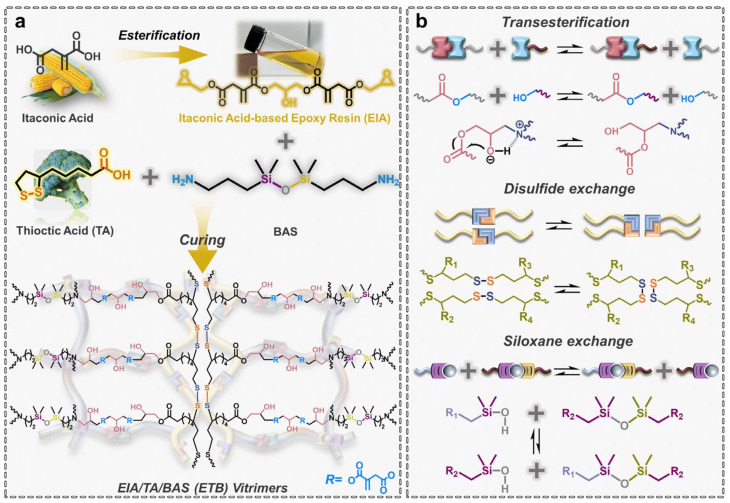
Synthesis of an IA-based covalent adaptable network with multiple dynamic bonds based on transesterification, disulfide exchange and siloxane exchange chemistry. Reproduced from ref. 138 with permission from Royal Society of Chemistry, copyright 2025.

Very recently, Qi *et al.* reported the synthesis of an covalent adaptable network with multiple dynamic bonds (Fig. 11).^138^ They synthesized an IA-based epoxy resin (EIA) which was reacted with thioctic acid (TA) and 1,3-bis(3-aminopropyl)-1,1,3,3-tetramethyl disiloxane (BAS) resulting in a cured material with dynamic bonds based on transesterification, disulfide exchange and siloxane exchange chemistry. The cured samples showed rapid exchange of dynamic bonds and intrinsic flame retardancy. Furthermore, the material can be degraded under basic conditions, making this material a very good example of a sustainable by design material. However, the use of epichlorohydrin in the synthetic procedure of the IA-based epoxy resin still leaves some room for further improvement in the synthesis of the materials.

### 3.6 Medical applications

In recent years, there has been growing interest to explore the utilization of IA within both biomedical and pharmaceutical research domains. In particular, IA-based monomers, such as octyl itaconate (OI) and DMI, have emerged as leading molecules for exploring therapeutic potential in a wide range of biomedical applications, *e.g.* diabetic wound repair^139,140^ inflammation,^141,142^ and anti-influenza agents to name a few.^143^ The exact mechanisms of the activity of monomeric IA-derivatives fall outside of the scope of our review, and have been described in detail in a recent work.^144^ However, in addition to IA-based monomers, also polymeric materials have been used for medical applications. A recurring strategy in recent biomaterials research involves the grafting of IA moieties *via* esterification onto pre-existing polymer backbones, such as hyaluronic acid,^145,146^ chitosan,^147^ or synthetic backbones. This modification introduces reactive unsaturated sites and functional carboxyl groups that enhance crosslinking potential and biological responsiveness. Notably, IA has also been grafted onto hydrophobic polymers like PDMS improving surface hydrophilicity and enabling biomedical functionalization.^148,149^ Such IA-grafted systems offer improved biological responsiveness, making them attractive for applications in tissue engineering, wound healing, and drug delivery platforms. IA has also been grafted on silica nanoparticles, serving as a crosslinker to covalently attach biomolecules.^150^ This approach enhanced the stability and accessibility of immobilized biomolecules, and it was used to improve the efficiency and sensitivity of biomolecular detection with the ELISA protocol, marking an example of the use of IA in biosensor applications.

Besides being grafted on pre-existing structures, IA can also be used as a building block for the preparation of hydrogels. One strategy involves the use of IA prepolymers which are subsequently incorporated into multicomponent networks with the use of acrylic monomers to yield composite^151,152^ or semi-interpenetrating polymer networks.^153^ Typically, it involves the condensation of IA with different diol moieties under relatively mild conditions (70–120 °C), and properties are tuned from the stoichiometry between the acid and the diol.^154–157^ Hydrogels prepared with this approach exhibit tunable mechanical and swelling properties, pH sensitivity and antibacterial activity.

**Scheme 17:**
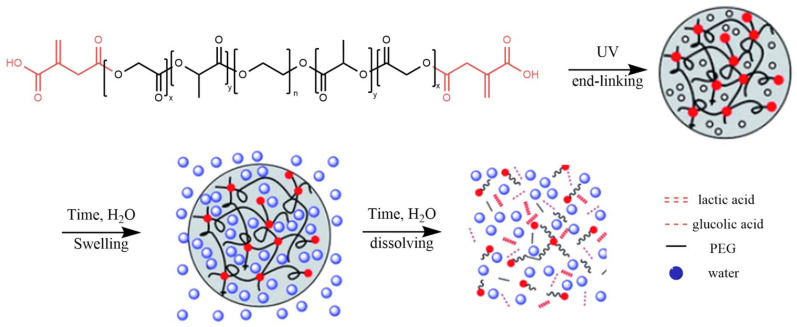
Example of an acrylate free IA-based hydrogel derived from lactic acid and ethylene glycol for biomedical applications. Reproduced from ref. 161 with permission from Royal Society of Chemistry, copyright 2016.

Finally, there are synthetic approaches of utilizing IA without any acrylates involved, to produce materials for biomedical applications. One strategy involves end capping of a polymer backbone with IA, trying to exploit either the double bond through radical crosslinking or the carboxylic groups. Existing studies have utilized Psluronic F127,^158^ PLA^159^ and PLA-PEG copolymers^160,161^ to prepare hydrogels with tunable swelling properties and a hydrolytic profile (Scheme 17). There is also a case of a non-isocyanate PU system based on vegetable oils, which was modified with IA to achieve pH responsiveness for an effective release of the hydrophobic drug lovastatin.^162^

Another strategy involves the preparation of a polymer backbone that includes a modified active molecule as part of the chain. Polyesters including chalcone^163^ and dicoumarol derivatives^164^ exhibited enhanced antibacterial properties while they were effectively used as scaffolds for tissue engineering. Composite materials can also be fabricated for this application, incorporating for example clay minerals, which can enhance the scaffold's mechanical properties and hydrophilicity.^165^ One key aspect of these materials is their ability to be hydrolyzed, as a result of the ester bonds, after they have served their purpose, but ester hydrolysis can also be exploited for drug release, for example by coating tablets with IA-based polyesters.^166^ Tuning the biodegradation rate of the polymer can help adjust the release rate of the encapsulated drug, but notably, given the immunoregulatory properties of IA, the polyesters themselves can be both the carrier and the active agent, as their hydrolysis yields unconjugated IA. This concept has been tested and proved in a number of studies, where the comonomers used for the synthesis of the polyester tuned the release profile of IA.^14,167,168^

## 4. Sustainability considerations

IA was mentioned in the first report by the US Department of Energy in 2004 as a top value added chemical.^169^ In 2010, Bozell *et al.* studied the progress made around these 12 high value chemicals.^170^ However, only 10 compounds of interest were selected, omitting IA based on the study's criteria, without undermining its potential. Though improvements had to be made at the time regarding its production and scalability, its potential as a bio-based platform molecule was already demonstrated. However, thorough life cycle analysis (LCA) is required to evaluate its benefit compared to those of fossil-fuel based or other bio-based molecules, or other unsaturated polyester resins. A complete LCA for IA production was performed by Rebolledo-Leiva *et al.* focusing on fermentation of wheat straw.^171^ A summary of the obtained values (baseline scenario) for the impact on global warming (GW), particle matter formation (PM), freshwater and marine eutrophication (FE), human toxicity (HT), land use (LU), fossil resource scarcity (FRS) and water consumption (WC) is provided in Table 3, together with the main contributing sections of IA production. Overall, these effects were mainly influenced by the inoculum and fermentation step followed by the pre-treatment step. Two optimization scenarios were explored with a steam explosion pre-treatment alone or combined with the use of 100% renewable energy. While first optimization reduced the global warming impact (from 14.33 to 12.72 kg CO_2_ eq.) and fossil fuel scarcity (from 4.15 to 3.47 kg oil eq.) slightly, it displayed a negative impact on freshwater use and eutrophication as well as global water consumption and human toxicity due to the steam explosion process. However, when 100% renewable energy sources were used, a significant reduction of impact was observed on GW (82%), PM (71%) and FRS (82%), showing the clear benefit in the environmental impact of IA production. Furthermore, other similar feedstocks could be envisaged for fermentation to IA,^171^ like brewer's spent grain, a waste biomass close in composition and reactivity to evaluate the impact of the feedstock on LCA results.^172,173^

**Table 3:** Summary of the impact and contributors for LCA of itaconic acid production and comparison with those from optimized bio-refineries^171^

Impact category	Baseline scenario	Unit	Main contributing sections (%)	Optimisation 1	Optimisation 2
GW	14.33	kg CO_2_ eq.	Inoculum and fermentation (40%), pre-treatment (35%)	12.72	2.64
PM	21.64	PM_2.5_	Inoculum and fermentation (42%)	21.83	6.22
FE	10.10	g P eq.	Inoculum and fermentation (55%)	11.66	1.27
HT	0.56	kg 1,4-DCB	Inoculum and fermentation (54%), recovery (21%)	0.63	0.07
LU	1.05	m^2^a crop eq.	Pretreatment (54%)	1.09	0.73
FRS	4.15	kg oil eq.	Pretreatment (37%)	3.47	0.76
WC	0.29	m^3^	Pretreatment (37%)	0.33	0.40

Kimbal *et al.* also studied the techno-economic feasibility of itaconic acid from woody biomass and more specifically pine wood on a 2400 tons per year scale and a market value around 3.25 € per kg (2011).^174^ LCA results showed a GW at 7.13 kg CO_2_ eq., a higher human toxicity (1.92 kg 1,4-DCB) but a lower land use (0.345 m^2^a) than the values reported by Rebolledo-Leiv *et al.* (Table 3).^171^ Compared to acrylic acid, they showed that using this technology, IA exhibited a higher environmental impact, in part due to the higher degree of optimization of traditional refineries compared to that of bio-refineries. As a comparison, the LCA of acrylic acid production from propylene using various fuels for steam generation performed by Petrescu *et al.* gave a GW ranging from 1.09 to 5.53 kg CO_2_ eq. per kg with the best results obtained when biomass was used as a fuel.^175^ HT was calculated between 18.74 and 57.16 kg 1,4-DCB, showing that IA production has a lower toxicity impact compared to that of acrylic acid. When obtained through a sustainable route *via* 3-hydroxypropionic acid, acrylic acid optimized production could theoretically reach a GW of 3.00 kg CO_2_ eq. per kg for a price around 1.29–1.52 $ per kg.^176^ The higher end of the GW range for fossil-based acrylic acid is in accordance with the conclusion of Adom *et al.* that showed a decrease in GHG emission of 53% for the bio-based acrylic acid (4.1 kg CO_2_ eq. per kg) compared to its non-renewable counterpart (8.4 kg CO_2_ eq. per kg).^177^ Montazeri *et al.* performed a *meta*-analysis of greenhouse gas (GHG) emissions and energy use regarding major bio-based chemicals and their petroleum-based counterparts.^178^ Data were based on the values presented in the above-mentioned studies. They found that compared to acrylic acid, IA production resulted in a 72% lower GHG emission when produced from corn carbohydrates, and it was 113% lower when produced from wood-based carbohydrates. Similarly, energy use was found to be 64% and 79% lower, respectively, compared to that with acrylic acid. It shows that in their case, IA production exhibited a much lower environmental impact.

Nuss *et al.* compared the LCA of polyitaconate from corn and wood biomasses and polyacrylate.^179^ They showed that the GWP and fossil energy demand (CED) were lower for wood-based (1.32 kg CO_2_ eq. per kg and 14.99 MJ eq.) and corn-based materials (2.19 kg CO_2_ eq. per kg and 24.8 MJ eq.) compared to those of the fossil-based material (2.74 kg CO_2_ eq. per kg and 70.58 MJ eq.), with wood biomass having the lowest impact in general. Furthermore, the GW calculations are even lower if the carbon temporarily sequestered within polymers are considered, leading to a GW of −0.13 and 0.74 kg CO_2_ eq. per kg for wood-based and corn-based polyitaconates, respectively. However, the use of both biomasses resulted in a higher land occupation (102–350-fold), water use (1.6–2.6-fold) and eutrophication (22.7–38.6-fold) compared to that of fossil-fuel based materials. As indicated above, the use of waste feedstocks should be investigated for such applications. Suriano *et al.* investigated the environmental profile of fossil-based UPE from isophthalic acid diluted in styrene and bio-based UPE from 2,5-furandicarboxylic acid, IA and propanediol (1,2 and 1,3) diluted in either butanediol diacrylate or dimethyl itaconate.^180^ The use of the bio-based resin allowed the reduction of GHG emission by 20.5% and the energy use by 46%. Further improvements using the IA-based reactive diluent allowed their reduction by another 27.5% and 44.3%, respectively, showing the positive impact of IA-based polymers and monomers. Similar to the finding of Nuss *et al.*, wood derived IA showed lesser impact on GHG emission and energy used compared to corn-based production. Finally, Montazeri *et al.* compared traditional fossil-based wood coating against bio-renewable ones from succinic acid and IA, 1,3-PDO, formulated with a soy-based resin.^181^ LCA showed that the bio-based coating had a reduced impact on ozone depletion (−31%), global warming (−40%), carcinogenic and non-carcinogenic toxicity (−29% and −74% respectively), as well as ecotoxicity (−38%) and fossil fuel depletion (−51%). However, it resulted in an increase in smog, acidification, eutrophication and respiratory effects according to the authors.

Despite the disparities of reported LCA results, inherent to the system boundary definition and the production technology used, data show a clear advantage in most criteria of IA-based monomers or polymers over their non-renewable counterparts (acrylates or styrene, Table 4). This improved environmental impact could even be improved as the production through biorefineries is to this day not as optimized as that through traditional production routes of fossil-based moieties. Wood biomass seems to be the lowest impact resource for IA production but as mentioned above, the use of waste biomass could allow further reduction of production's environmental impact, less competition in soil use and reduction of waste production.

**Table 4:** Summary of GW impact for itaconic acid or itaconic acid-based materials

Reference	Material (LCA precisions)	GW impact (kg CO_2_ eq.)
Rebolledo-Leiva^171^	Itaconic acid (base LCA)	14.33
	Itaconic acid (pre-treatment optimisation)	12.72
	Itaconic acid (pre-treatment optimisation and use of renewable energy)	2.64
Kimball^174^	Itaconic acid	7.13
Petrescu^175^	Acrylic acid (using various fuels for steam generation)	1.09–5.53
Adom^177^	Fossil-based acrylic acid (natural gas as fuel)	8.7
	Bio-based acrylic acid (natural gas as fuel)	4.1
Bhagwat^176^	Bio-based acrylic acid	3.00
Nuss^179^	Polyitaconate (wood-based)	1.32
	Polyitaconate (corn-based)	2.19
	Polyitaconate (fossil-based)	2.74

## 5. Conclusions and future perspective

This article comprehensively reviews research conducted on the synthesis and application of (poly)condensation chemistry to prepare monomers and polymers from itaconic acid. For the latter, the focus is placed on unsaturated polyesters, but also saturated polyesters and poly(ester amide)s are covered. Work on the synthetic procedures (classical chemical and enzymatic) is discussed followed by highlighting applications where these bio-based monomers and polymers are currently utilized. The conjugated double bond located at the *exo*-position makes these compounds suitable for a large variety of applications, such as thermal- and UV-curing unsaturated polyesters. They therefore represent more sustainable alternatives to malic acid-based unsaturated polyesters and polyester (meth)acrylates with applications in composites, (UV-curing) coatings, elastomers and UV-curing additive manufacturing. In addition, itaconic acid-based materials recently drew attention in other fields, such as covalent adaptable networks (especially vitrimers) or biomedical applications, where the low toxicity is beneficial. For vitrimers, the trifunctional nature of itaconic acid allows for the implementation of several types of dynamic covalent networks with a focus on transesterification chemistry. Finally, the sustainability of the itaconic acid-based materials is discussed by comparing life cycle analysis results in the field, which show improved values in comparison with those of petrochemical building blocks.

The amount of novel work over the last ten years, also in new application areas, shows the future potential for itaconic acid-based materials. One of the main reasons for this is that itaconic acid is more than a bio-based drop-in solution, as it was never available on industrial scale derived from petrochemical feedstock. This has two major advantages: first, the trifunctional nature enables novel polymer architectures, offering the potential for improved properties and performance in the final materials, which could be complementary or superior to those of established polymeric materials. Second, this improved performance can justify higher prices, which is an advantage that drop-in solutions such as succinic acid or 1,4-butanediol do not have. This type of bio-based building block can only compete when their costs are close to or lower than the price of their petrochemical counterparts.

However, despite the significant advances and the high potential, several challenges remain. Itaconic acid is commercially available at competitive prices of around 2 € per kg and is already used on an industrial scale in polycondensation reactions or as a comonomer in radical polymerizations. Other itaconic acid-based building blocks, such as itaconic anhydride and various diesters, on the other hand are still not commercially available on an industrial scale. Enlarging the scope of these building blocks could lead to further industrial applications of itaconic acid-based materials. In addition, the synthesis of amide-derived monomers and polymers that retain the conjugated double bond is only possible in low yields and through laborious cumbersome and non-scalable synthetic procedures. Novel approaches that are also feasible on a multigram or kilogram-scale would be of high scientific value to be able to study the properties and potential of these amide-based materials derived from itaconic acid. Also, dendrimeric polyesters derived from itaconic acid are of high interest, as this could lead to a significant reduction of viscosity compared to that of their linear counterparts with similar molecular mass. To the best of our knowledge this class of polyesters have not been reported to date, as standard esterification processes lead to transesterification reactions and eventual loss of branching.

Finally, it should be noted that the commercial biotechnological production of itaconic acid does not, in itself, guarantee a reduced environmental footprint. Even though the life cycle assessments discussed herein look promising, the data availability is limited, especially for large scale commercial sources. Here, more work is needed to fully evaluate the sustainability of itaconic acid and materials derived from it. This will further fuel the journey of this interesting molecule towards a significant role in a sustainable bioeconomy with a real economic impact.

## Conflicts of interest

There are no conflicts to declare.

## Data Availability

No primary research results, software or code have been included and no new data were generated or analyzed as part of this review.
